# The RAFT-Mediated Synthesis of Poly(styrene-*co*-maleic acid) through Direct Copolymerization of Maleic Acid

**DOI:** 10.1021/acs.macromol.5c01372

**Published:** 2025-07-31

**Authors:** Michael-Phillip Smith, Lauren E. Ball, Bert Klumperman

**Affiliations:** Department of Chemistry and Polymer Science, 26697 University of Stellenbosch, Private Bag X1, Matieland 7602, South Africa

## Abstract

This work describes the reversible addition–fragmentation chain-transfer (RAFT)-mediated synthesis of poly­(styrene-*co*-maleic acid) (SMA). Various comonomer feeds were investigated to acquire tunable nonalternating SMA. The isolated copolymer constituted unique triad distributions, affording dissimilar physical properties compared to those of commercially available alternatives (SMA2000). The solubilization of synthetic DMPC and POPC lipid vesicles, using copolymers with variable composition, was investigated to elucidate the effect of triad distribution on SMA lipid particle (SMALP) formation. It was revealed that copolymers with similar overall amphiphilicity (composition of hydrophobic and hydrophilic repeat units in the copolymer) exhibit dissimilar interactions with a lipid bilayer as a result of different comonomer triad distributions, i.e., SSS, SSM, and MSS, and MSM triads. This work emphasizes the importance of the distribution of hydrophobic and hydrophilic comonomer units along the SMA backbone.

## Graphical abstract

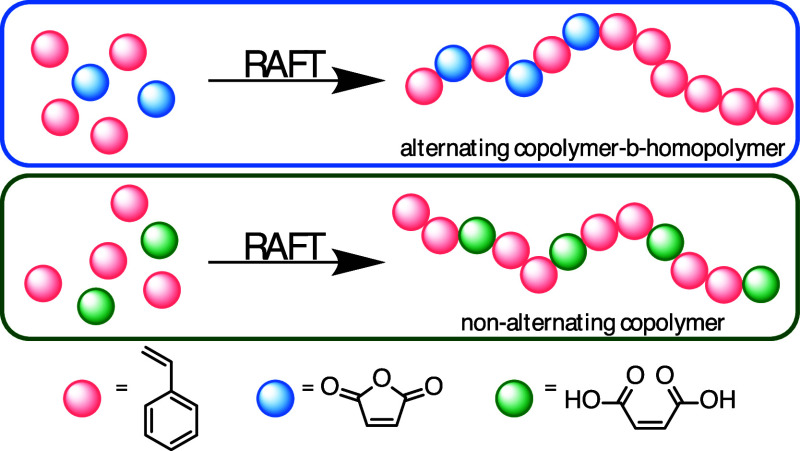

## Introduction

Poly­(styrene-*co*-maleic acid) (SMA) is a desirable copolymer system that finds utility in drug delivery^1,2^ and membrane protein (MP) solubilization,^3^ among other applications. For the solubilization of MPs from their native lipid environment, the SMA composition has a significant impact on solubilization efficiency. An optimal ratio of hydrophobic to hydrophilic comonomer units (S:MA = 2:1) is required to facilitate the required copolymer interactions at the lipid–water interface.^4,5^ A large research effort has focused on synthesizing copolymers that mimic this optimal amphiphilicity through direct copolymerization of varying comonomer polarities^4^ or via postpolymerization functionalization of maleic anhydride moieties with hydrophobic functionalities.^6–8^

In previous reports, SMA was afforded via the copolymerization of styrene (S) and maleic anhydride (MAnh) to generate poly­(styrene-*co*-maleic anhydride) (SMAnh). Subsequently, SMAnh can be hydrolyzed under basic aqueous conditions to obtain poly­(styrene-*co*-maleic acid) (SMA) (Figure 1A).^9^ This indirect process is time-consuming due to multiple reaction steps and associated workup procedures. Conversely, this current study explores an alternative route to synthesize SMA via direct copolymerization of styrene and maleic acid (MA). To our knowledge, there is a single publication concerning the conventional radical copolymerization of styrene and maleic acid conducted by Świtała-Żeliazkow.^10^

**Figure 1:**
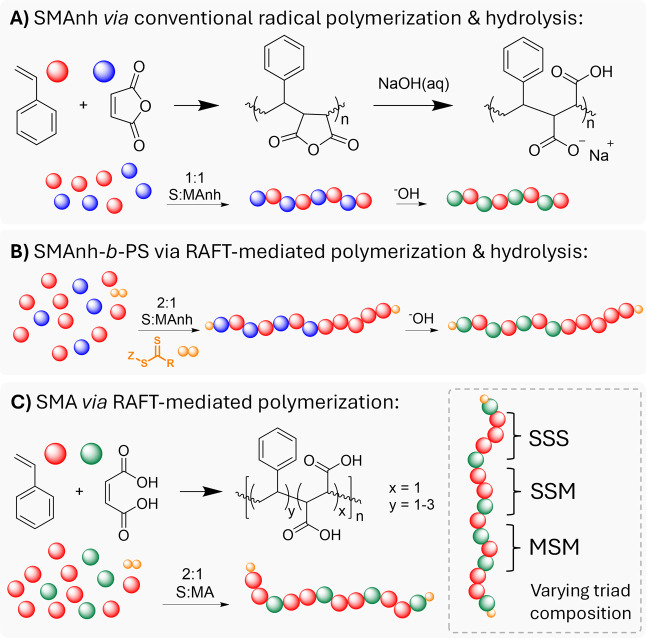
Comparison of copolymers synthesized via different RAFT-mediated approaches. (A) S (red dots) and MAnh (blue dots) in a 1:1 ratio afford alternating SMAnh that can be hydrolyzed in basic aqueous media to form SMA 1:1 (where MA = green dots). (B) When copolymerizing S and MAnh via RAFT-mediated polymerization in a 2:1 ratio, a block copolymer is synthesized. (C) Utilization of an acceptor monomer that is less electron-deficient to afford nonalternating SMA via direct RAFT-mediated copolymerization.

Unlike the direct copolymerization of S and MA, the copolymerization of S and MAnh has undergone thorough investigation over the decades. Nonalternating SMAnh can be synthesized via batch-wise radical copolymerization at low monomer conversion (low monomer consumption during copolymerization) with variable copolymer composition (0–50 mol % MAnh), which is controlled through adjustment of the comonomer feed composition.^11,12^ However, composition drift readily occurs during the copolymerization and the copolymer acquired has the typical *Đ* of >1.5 for a conventional radical polymerization.^9^ Nonalternating SMAnh with a narrow chemical composition distribution (CCD) but still a *Đ* of >1.5 can be synthesized in a more facile and scalable manner via radical copolymerization in a continuous stirred tank reactor (CSTR).^13^ As a result of the steady-state operation of a CSTR system, composition drift is effectively suppressed and the resulting CCD is solely governed by the statistical nature of a radical copolymerization reaction.^13,14^

Since the 1990s, controlled radical polymerization techniques have been developed and optimized for many (co)­monomer systems.^15^ Reversible addition–fragmentation chain-transfer (RAFT)-mediated polymerization is an effective tool for synthesizing low *Đ* SMAnh but has not been employed for the copolymerization of S-MA systems.^3,5,9,16^ It has been shown in previous works that the S-MAnh system constitutes a strongly alternating comonomer pair as a result of the electron-rich (donor) and electron-poor (acceptor) properties of S and MAnh, respectively.^17^ As a consequence, the RAFT-mediated copolymerization of S and MAnh leads to a copolymer that is highly alternating in nature (Figure 1A). If an excess of S is present in the monomer feed, a block copolymer is formed where the first block is composed predominantly of alternating S-MAnh sequences with the second block constituting the styrene homopolymer (Figure 1B).^18,19^ To synthesize a RAFT-mediated nonalternating SMA, MAnh could be substituted with a less electron-deficient monomer. Careful monomer substitution would afford a RAFT-mediated copolymerization with diminished alternating character and therefore afford SMA with tunable monomer distribution and low *Đ* (Figure 1C).

This study investigated MA as an acceptor monomer to afford nonalternating SMA. A computational analysis was conducted to determine the respective electron occupancies of the vinyl bonds in MA and MAnh to investigate the relative acceptor behavior. Thereafter, a RAFT-mediated methodology was developed for the S-MA system. Various comonomer feed compositions were explored to determine the reactivity ratios of the comonomers. A library of copolymers with varying copolymer compositions (varying ratios of S:MA) was synthesized via RAFT-mediated copolymerization and characterized via nuclear magnetic resonance (NMR) spectroscopy and thermogravimetric analysis (TGA). The solution properties were examined via titration of the copolymers with acidic or divalent cations. The copolymers were thereafter utilized to solubilize synthetic lipid vesicles (1,2-dimyristoyl-*sn*-glycero-3-phosphocholine (DMPC) and 1-palmitoyl-2-oleoyl-glycero-3-phosphocholine (POPC)) to assess the effect of copolymer composition and microstructure on the solubilization efficiency.

## Experimental Methods

### Materials

All chemicals were purchased from Merck Life Sciences unless otherwise stated. Maleic anhydride (99%) was recrystallized from distilled chloroform (40 °C), filtered, and thereafter dried *in vacuo* at 40 °C. Maleic acid (≥99%) was utilized as received. Styrene (≥99%, stabilized with *tert*-butylcatechol) was passed through an aluminum oxide column thrice to remove the inhibitor (30 min prior to copolymerization). Azobis­(isobutyronitrile) (AIBN, 95%) was recrystallized from anhydrous methanol, filtered, and thereafter dried *in vacuo*. SMA2000 was obtained from Cray Valley, USA, and underwent alkaline hydrolysis, dialysis, and lyophilization prior to use. Anhydrous 1,4-dioxane (99.8%), anhydrous *N*,*N*-dimethylformamide (99%), HCl (32%), and NaOH (pellets for analysis) were utilized as received. 3500 MWCO SnakeSkin dialysis tubing (purchased from Thermo Fisher Scientific) was utilized as received.

### Generalized Synthetic Procedure for the Copolymerization of Styrene and Maleic Acid

RAFT agents were synthesized according to work published by Klumperman and coworkers.^20^ In a typical S-MA (1:1) copolymerization, an oven-dried flask was charged with styrene (0.75 g, 7.20 mmol, 50 equiv), maleic acid (0.84 g, 7.20 mmol, 50 equiv), AIBN (5.0 mg, 0.03 mmol, 0.2 equiv), DMF (as an internal standard, 6 mg), and RAFT agent (0.14 mmol, 1.0 equiv). These components were dissolved in 1,4-dioxane (5.55 mL) to afford a copolymerization mixture at 30 w/v%. The mixture was thereafter sparged with dry argon for 30 min, and a t_0_ sample was collected with a degassed syringe. The flask was immersed in a preheated oil bath (temperature dependent on the experiment) for 30 h. The copolymer was thereafter isolated via precipitation in 1 M HCl solution. The copolymers were characterized by using NMR spectroscopy and size exclusion chromatography (SEC). The SMA was purified further by resuspension in deionized (DI) water/acetone (∼1:1, pH = 12–13) and dialyzed against DI water/acetone (0.8:0.2) for 24 h, followed by DI water for up to 48 h. The copolymer was dried via lyophilization and subsequently utilized in TGA, titration, and solubilization experiments.

### Characterization

Nuclear magnetic resonance (NMR) spectroscopy was undertaken using a Bruker Ascend spectrometer, 400 or 600 MHz. All samples were prepared in acetone-*d*_6_ (99.9%). Kinetic samples (0.1 mL) were diluted in 0.4 mL of deuterated solvent and analyzed via ^1^H NMR spectroscopy (400 MHz) to determine monomer conversion. Purified copolymer samples (0.1 g) were dissolved in 0.7 mL deuterated solvent and analyzed via ^1^H NMR spectroscopy and quantitative ^13^C NMR spectroscopy (600 MHz) to characterize copolymer composition and styrene-centered triad distribution, respectively. Quantitative ^13^C NMR spectroscopy was conducted on a Bruker system with C13IG experimental program with 3300 scans and a 15 s D1 delay, at a temperature of 298 K and zgig pulse program. Triads were determined according to ppm ranges reported in the literature^21^ and were confirmed by comparison to ^1^H NMR copolymerization conversion data and ^13^C NMR data of the isolated copolymer by back calculation (see Supporting Information, pg. 2).^22^ All data were processed using MestreNova 11.3 software.

Size exclusion chromatography (SEC) was used to characterize the molecular weight distribution and *Đ* values of the copolymers. Samples were prepared at a 2 mg·mL^–1^ in 2 mM LiBr-stabilized DMF (for HPLC, ≥ 99.9%) for 24 h before analysis. All samples were filtered with 0.45 μm PTFE filters. Samples were analyzed using an Agilent 1260 HPLC instrument, constituting an autosampler, a quaternary pump, a thermostated column compartment (60 °C), a differential refractometer (50 °C), and a diode array UV detector (320 nm). Columns utilized were Agilent PLgel Mixed-C (5 μm), specifically one guard column (50 × 7.5 mm i.d.) and two analytical columns (300 × 7.5 mm i.d.). A flow rate of 1.0 mL·min^–1^ and a sample injection volume of 100 μL were used. The SEC system was calibrated using PMMA standards (800–2 200 000 g·mol^–1^) and SMAnh calibration standards (600–90 000 g·mol^–1^, synthesized via RAFT-mediated copolymerization). Molecular weight and *Đ* analysis were conducted using WinGPC UniChrom, Build 5350 software.

Thermogravimetric analysis (TGA) was conducted using a TA Instruments Q500 system under a nitrogen gas purge (flow rate 40.0 mL·min^–1^) using aluminum sample pans at a ramp rate of 10 °C·min^–1^ to 600 °C. Samples were predried *in vacuo* overnight at 80 °C. Approximately 5 mg of sample (ring-opened and neutralized to pH 7) was utilized per analysis. Data were analyzed and interpreted in TA Universal Analysis and OrginPro 8.5.

Dynamic light scattering (DLS) analyses were conducted using a ZetaSizer 1000 HSa (Malvern Instruments, Malvern), fitted with a 4 mW He–Ne laser operating at a wavelength of 633 nm and a scattering angle of 90°. Analyses were conducted using ZetaSizer Software 8.02 and the data were processed using OriginPro 8.5 software.

Turbidimetry experiments were conducted by using a Thermo Fisher Scientific Multiskan SkyHigh microplate spectrophotometer fitted with a xenon flash lamp and a monochromator with a bandwidth of less than 2.5 nm. Measurements were obtained at 600 nm, with samples incubated at 25 °C during analysis. For titration experiments, copolymer stock solutions were prepared at 10 mg·mL^–1^ in Tris HCl buffer (50 mM, for M^2+^ titrations) or DI water (for acid titrations). The copolymer solution (300 μL) was added to a quartz cuvette, an aliquot of Mg^2+^/Ca^2+^ solution (10 μL, 0.1 M) was added, and the optical density was measured. For acid titrations, the copolymer solution (1 mL) was titrated with HCl (10 μL aliquots, 0.5 M), the pH was measured using a calibrated pH meter (Mettler Toledo, FiveEasy model), and thereafter the solution was transferred to a quartz cuvette and the optical density measured. For experiments relating to the solubilization of lipid vesicles, POPC or DMPC vesicles (10 mg·mL^–1^) were freshly prepared via sonication (0.5–1 h) of the lipid suspension in Tris HCl buffer (50 mM). The lipid vesicle solution (10 μL) was diluted in Tris HCl buffer (990 μL) and the hydrodynamic diameter of the particles was assessed via DLS. Thereafter, 300 μL of the POPC/DMPC vesicle solution (10 mg·mL^–1^) was added to a quartz cuvette and incubated at 25 °C in the spectrophotometer prior to the addition of the copolymer solution (300 μL, 50 mg·mL^–1^), at which point the optical density was measured every 3 s for 5 min. All turbidimetry experiments were performed in triplicate and the data were processed using OriginPro 8.5 software.

### Computational Method

#### RAFT Copolymerization

The computational work was completed on the Jaguar module within the Schrödinger Suite (2021-4),^23^ which was accessed through the Maestro graphical interface.^24^ The structures were minimized utilizing density functional theory at the B3LYP level,^25–28^ with the 6-31+G* basis set.^29–32^ The “check_min = 1” function in Jaguar within Maestro was employed to verify that all of the optimized geometries represented true energy minima. All calculations were conducted with unrestricted spin. The THF solvent environment was utilized with the conductor-like polarizable continuum model (CPCM).^33,34^ All basis sets were obtained from the Basis Set Exchange.^35–37^

#### Monomer Properties

The same conditions were utilized, except the investigation was conducted at the M06-2X^38–40^ and 6-31+G** levels.^29–32^

### Polymerization Model Determination

Reactivity ratios were determined using a nonlinear least-squares (NLLS) procedure based on the IUPAC-recommended method that was recently published.^41^ The procedure was carried out for the terminal model (TM) and for the restricted penultimate unit model (rPUM), where homopropagation of maleic acid is excluded from the equations. Where the IUPAC-recommended method performs calculations based on copolymer composition, in the current calculations, triad fractions were used as input data. The NLLS procedure entailed the calculation of the sum of squares for each reactivity ratio pair over a selected interval of values. During the calculation for each individual reactivity ratio pair, numerical integration was employed to account for composition drift during each individual experiment.

## Results and Discussion

Electron-rich (donor) and electron-deficient (acceptor) comonomer pairs have been subject to many studies over the years.^17,20,42^ The more extreme a monomer is toward these classifications (either electron-rich or -deficient), the more likely the comonomer pairs are to partake in alternating copolymerizations.^43^ A textbook example of this is styrene (electron-rich) and maleic anhydride (electron-deficient). In previous studies, it has been shown that the copolymerization follows the penultimate model.^17^ To decrease the alternating tendency compared to the S-MAnh copolymerization, a less electron-deficient comonomer was pursued in this study, namely, maleic acid (MA). It was hypothesized that the electron density of the vinyl bond would differ as a result of the varying functional groups between the two monomers.

Analysis of the Wiberg bond index (a measure of bond order, i.e., singly or doubly bonded atoms)^44–47^ indicates that MA has a stronger vinyl bond than MAnh, which in turn describes a bond with higher electron density in MA than in MAnh (Table 1). This was further confirmed by the Natural Bond Order (a measure of the strength of a chemical bond)^48,49^ occupancies being larger for MA (Table 1). Both computational results confirm that the electron density found in the MA vinyl bond is greater than that of MAnh. Therefore, MA is less of an electron-deficient monomer than MAnh and is expected to result in a copolymerization with a weaker alternating tendency than its anhydride counterpart when copolymerized with S.^43^

**Table 1:**
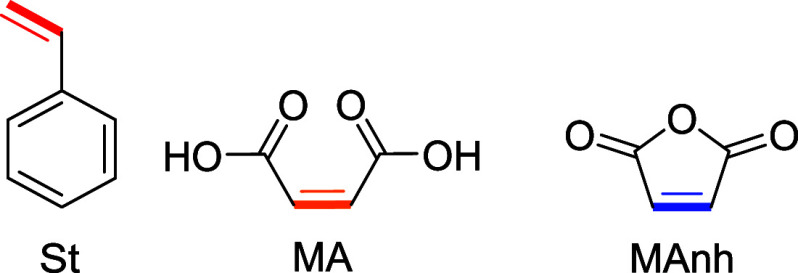
Electronic Monomer Properties^a^

Property			
Natural bond order σ bond (occupancy/energy (kcal/mol))	(0.99486/ −0.81841)	(0.99020/–0.87196)	(0.99015/–0.90351)
Natural bond order π bond (occupancy/energy (kcal/mol))	(0.97357/ −0.32322)	(0.95639/–0.36577)	(0.92986/–0.40376)
Wiberg bond index	1.9174	1.8947	1.8442

a The calculated data pertain to the highlighted bonds.

Reaction condition screening was conducted to determine the optimal polymerization temperature and solvent (Table 2) initially using a dithiocarbamate (DTC) chain transfer agent (CTA) as this CTA has been shown to provide optimal control for the copolymerization of S and MAnh (Figure 2).^20,50^ 1,4-Dioxane resulted in a higher monomer conversion (α), in comparison to the more polar solvent (DMF).^51^ A temperature of 70 °C proved to be optimal for the copolymerization, with higher temperatures resulting in the isomerization of maleic acid to fumaric acid and lower temperatures resulting in lower conversion (Table 2).

**Table 2:** Experimental Conditions for Synthetic Protocol Optimizations for the Copolymerization of MA and S^a^

Temp (°C)	Solvent	RAFT CTA	α^S^ 30 h (%)	α^MA^ 30 h (%)	Isomerization (%)	AIBN: CTA
60	DMF	DTC	15	1	0.5	0.2
70	DMF	DTC	19	7	1	0.2
80	DMF	DTC	20	8	4	0.2
70	Dioxane	DTC	34	32	N.D.	0.2

a (N.D. = not determined). S:MA:CTA = 50:50:1.

**Figure 2:**
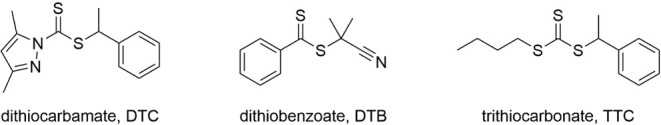
Screened CTAs during this study. Each CTA class has been utilized to copolymerize SMAnh:1—dithiocarbamate, 2—dithiobenzoate, and 3—trithiocarbonate.

CTA screening was conducted to determine the optimal Z-group for the SMA copolymerization (Figure 2).^20^ Inspection of the kinetic profiles highlights that only two CTAs successfully mediated the copolymerization (DTB and TTC, Figure 3).

**Figure 3:**
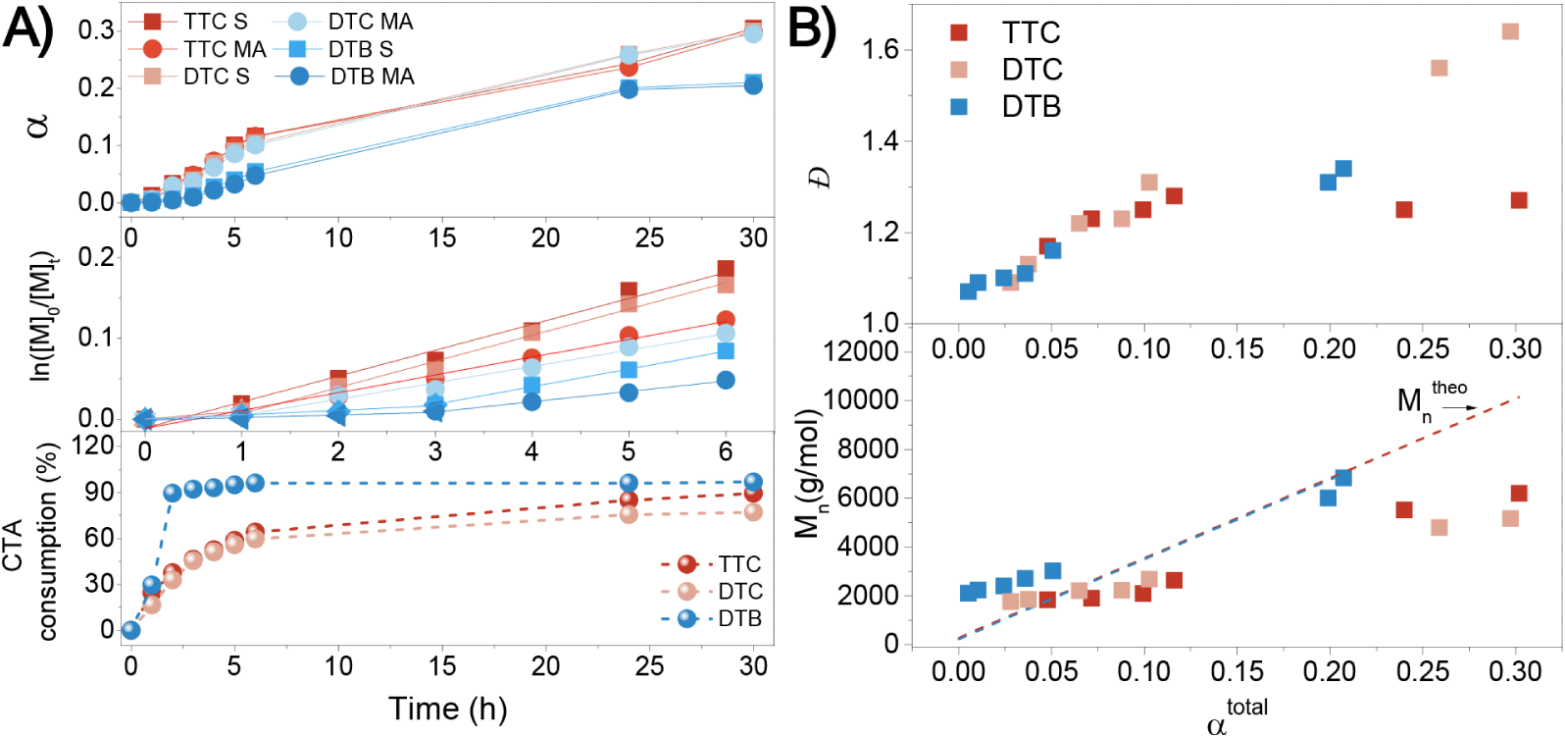
SMA copolymerization at a monomer feed of 60:40 (S:MA) with different CTAs, namely, DTC, DTB, and TTC in dioxane, S:MA:CTA = 180:120:1. (A) From top to bottom, monomer conversion, ln­([M]_0_/[M]_*t*_), and CTA consumption (%) as a function of time. (B) *M*_n_ and *Đ* as a function of monomer conversion (α). Solvent: dioxane.

The DTC-mediated copolymerization of S and MA led to an overall conversion, α = 0.29 after 30 h (Figure 3A, **top**), and predominantly, S was incorporated at a faster rate than that of MA. The dispersity was shown to gradually increase as a function of overall monomer conversion to a value of 1.64 at α = 0.30, which was the maximum conversion reached after 30 h (Figure 3B). This might be a result of the slow conversion of the CTA during the copolymerization, as the final conversion of DTC after 30 h was determined to be α = 0.77 (Figure 3A, bottom). In comparison, DTB was fully converted within 5 h, which equates to circa an overall monomer conversion α = 0.05 (Figure 3A). As a result, SMA synthesized using DTB exhibited lower *Đ* (1.34, Figure 3B, top), albeit at lower overall monomer conversion, and also in the case of DTB, a gradual increase in *Đ* is observed as a function of time. As is common for dithiobenzoates, there was a significant initialization period observed in the copolymerization for the first 3 h (Figure 3A). This behavior is well-known for the DTB-mediated synthesis of SMAnh,^52^ and thus, it is no surprise that it is also observed with this derivative system. As a result of this long initialization period, the copolymerization reaches a low α of only 0.21 after 30 h (Figure 3A, top). TTC was found to be the optimal CTA for the RAFT-mediated synthesis of SMA. TTC-mediated copolymerizations attained higher α (0.30, Figure 3A, top) with S incorporated at a slightly faster rate than MA. Although the TTC exhibited slower consumption compared to DTB (Figure 3A, bottom), it provided good control over the copolymerization as SMA with moderately low *Đ* (1.27, Figure 3B, **top**) was obtained and *M*_n_^SEC^ evolved linearly with increasing α. *M*_n_^SEC^ deviated from *M*_n_^theo^ at higher monomer conversion, which may be a result of the gradual CTA consumption throughout the copolymerization. Based on the optimization experiments, subsequent SMA copolymerizations were conducted at 70 °C in 1,4-dioxane using TTC as the RAFT agent.

The data in Figure 3 are further confirmed via computational analysis (Figure 4), where it is noted that the energy required for an MA radical to react with TTC (forming an intermediate radical, Δ*G* = 16.6 kcal·mol^–1^) is larger than that of for the respective reaction for MAnh (Δ*G* = 13.9 kcal·mol^–1^). The inefficient interaction of the macro-CTA with the monomer could further explain the deviation of *M*_n_^SEC^ from *M*_n_^theo^. Computational data further substantiate this hypothesis based on the differing associated Gibbs free energies (Δ*G* = 18.2 kcal·mol^–1^ and Δ*G* = 16.6 kcal·mol^–1^ for MA and Δ*G* = 16.9 kcal·mol^–1^ and Δ*G* = 13.9 kcal·mol^–1^ for the MAnh systems, Figure 4).

**Figure 4:**
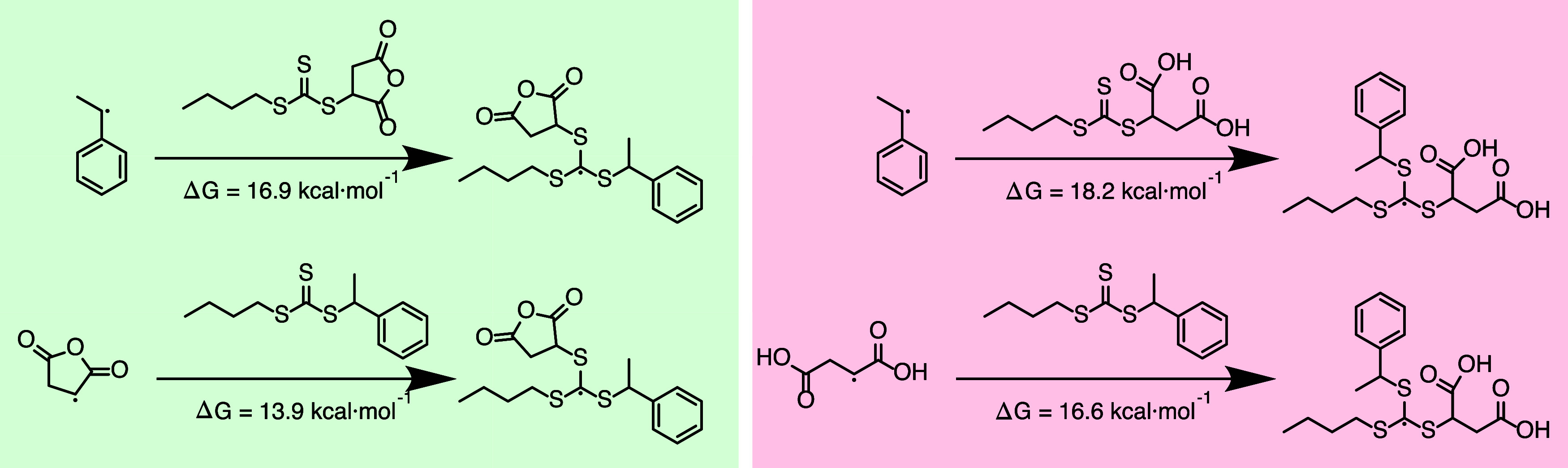
Summary of computational analyses of the RAFT-mediated synthesis of SMAnh and SMA. Gibbs free energies (kcal·mol^–1^) as calculated for the reaction of the TTC with S, MAnh, and MA to form an intermediate radical species.

Owing to the low α obtained *vide supra*, the copolymerization kinetics of SMAnh and SMA systems were compared, where the 1:1 (S:MAnh) copolymerization was significantly faster^20^ than the respective SMA copolymerization. A computational investigation highlighted that a possible cause for this lower rate of copolymerization could be the energy required for an MA propagating radical to react with an S monomeric unit (Δ*G* = 14.3 kcal·mol^–1^), which is larger than the corresponding reaction with a MAnh radical (Δ*G* = 12.7 kcal·mol^–1^) (Figure S6). Coupled with the notion that MA is less of an acceptor monomer than MAnh (Table 1), this could explain the slower copolymerization of SMA compared to that of SMAnh.

The SMA system was thereafter copolymerized at varying comonomer feed compositions to determine the reactivity ratios of this copolymer system (Table 3). NMR spectroscopic analyses suggested that there is good agreement between the copolymer composition of the isolated copolymer and the copolymer composition based on comonomer conversion (Table 3). As a result, it can be stated tentatively that limited side reactions occurred during the copolymerization and thus all consumed monomers were incorporated in the isolated copolymer. Therefore, the facile determination of the targeted SMA composition is accessible by simply tracking individual comonomer conversions via ^1^H NMR spectroscopy.

**Table 3:** Comonomer Feed Composition and Its Impact on Triad Composition Data of Isolated Copolymers

**f**_MA_	SSS fraction^a^	SSM + MSS fraction^a^	MSM fraction^a^	F_MA_ ^b^	**F**_MA_ ^a^
0.70	0.04	0.24	0.71	0.49	0.49
0.60	0.02	0.30	0.68	0.48	0.47
0.50	0.10	0.41	0.48	0.44	0.43
0.40	0.14	0.44	0.42	0.39	0.40
0.33	0.23	0.48	0.29	0.33	0.33
0.30	0.28	0.51	0.22	0.32	0.32
0.25	0.35	0.49	0.16	0.27	0.28
SMA2000	0.19	0.67	0.14		0.32 (lit = 0.33)

a Based on ^13^C NMR spectroscopy.

b Based on monomer ^1^H NMR spectroscopy conversion data. Comparison of data accuracy summarized in Figure S1.

In previous work, the relationship among comonomer feed composition, copolymer composition, and associated monomer triad distribution for the copolymerization of SMAnh has been established.^11,21,22^ Based on triad distributions and the independently measured average propagation rate constant as a function of comonomer feed composition, the rPUM was established as best describing the copolymerization kinetics.^11,17^ The associated reactivity ratios of the SMAnh system were determined as *r*_SS_ = 0.023 and *r*_MAnhS_ = 0.15 (60 °C, CSTR in methyl ethyl ketone).^14^ In the current study, the reactivity ratios of the SMA system were determined via compiling the composition and triad data (Table 3) and subjecting it to nonlinear least-squares fitting (*r*_SS_ = 0.464 and *r*_MAS_ = 0.456) (Figure 5). Since these reactivity ratios are the same within experimental error (*r*_S_ = *r*_SS_ = *r*_MAS_), it can tentatively be postulated that the S-MA comonomer pair obeys the restricted terminal model (rTM). This conclusion is based on the copolymer composition and triad distribution data. Additional research using, for example, pulsed laser polymerization is required to determine the penultimate unit effect on the average propagation rate constant.^17^ It is known that for many radical copolymerization systems, copolymer composition and triad distributions can adequately be described by the TM, whereas the PUM is required to also describe the propagation rate constant versus comonomer feed composition.^53^ Nevertheless, it can be concluded that the direct copolymerization of S and MA offers a facile alternative to the synthesis of nonalternating SMA in comparison to the route via SMAnh and subsequent hydrolysis.

**Figure 5:**
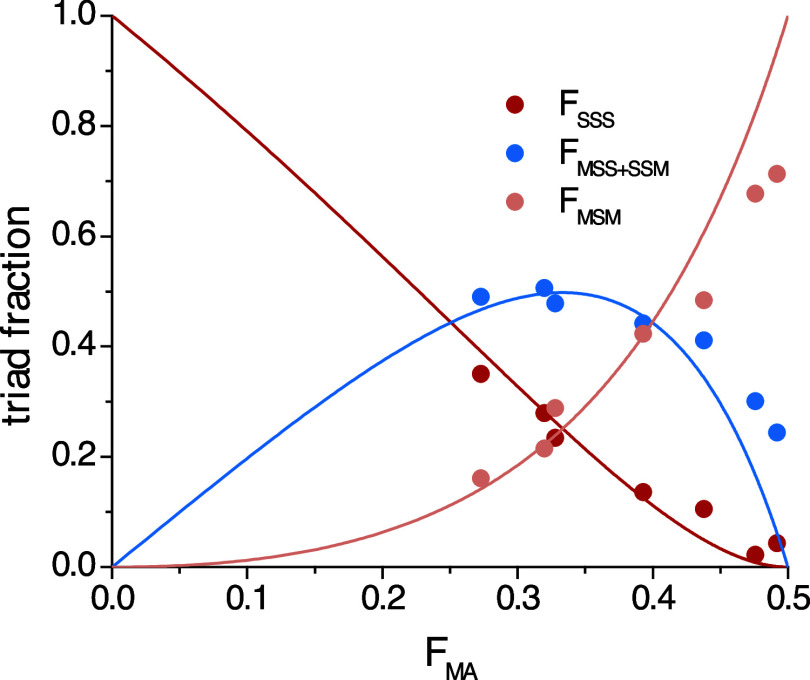
Triad distribution as a function of the S content in the isolated copolymer. Drawn curves are based on the rTM value with *r*_S_ = 0.46.

All triad assignments were determined based on ^13^C triad signal assignment (see Supporting Information, pg. 2) and compared to the copolymer composition calculated using comonomer conversion data, or via ^13^C NMR spectroscopic analysis of the isolated copolymers.^21,22^ All methods produced similar results, confirming that the triad description acquired was reliable (Figure S2).

Utilizing different comonomer feeds, SMA with varying compositions was synthesized for further analysis (Table 4). Sample SMA 2:1 and SMA2000 were analyzed via thermogravimetric analysis (TGA) to compare the physical characteristics of the copolymers with seemingly similar F_MA_ values of 0.33 and 0.32, respectively. It was found that the copolymers generated dissimilar TGA curves (Figure 6). On inspection, it can be noted that the SMA 2:1 copolymer has a different triad distribution (SSS = 0.27, SSM and MSS = 0.46, MSM = 0.27) in comparison to the commercial SMA2000 (SSS = 0.19, SSM and MSS = 0.67, MSM = 0.14). This difference in triad distribution, i.e., polymer backbone microstructure, could explain the difference in TGA profiles. In 1993, Klumperman and coworkers filed a patent regarding a base-catalyzed modification reaction of SMAnh.^54^ In this invention, it was stipulated that spirodilactonization with the concomitant release of carbon dioxide can occur within the backbone of the SMAnh copolymer. This reaction occurs specifically on MSM fractions in SMAnh copolymers at elevated temperatures (200–270 °C). Noting that SMA 2:1 has larger fractions of the MSM triad than SMA2000 (0.27 vs. 0.14, respectively), it is plausible that the mass loss for SMA 2:1 in the 200–300 °C range corresponds to a greater prevalence of the spirodilactonization reaction (Figure 6). The TGA data is in agreement with the calculated differences in the triad fractions for SMA 2:1 and SMA2000 and confirms that the SMA 2:1 copolymer possesses a different monomer sequence distribution, despite the nearly identical overall chemical composition.

**Table 4:** Copolymer Library of SMA Derivatives with Varying S:MA Ratios

Copolymer	*f*_MA_	F_MA_ ^a^	*M*_n_^theo^ (g·mol^–1^)	*M*_n_^SEC^ (g·mol^–1^)	*Đ*
SMA 1:1	0.50	0.45	7900	5290	1.33
SMA 1.5:1	0.40	0.40	7780	5090	1.20
SMA 2:1	0.33	0.33	7710	4730	1.30
Hydrolyzed SMA2000	-	0.32^b^	-	4200	1.90

a Determined with comonomer polymerization ^1^H NMR spectroscopy conversion.

b Based on triad distributions from ^13^C NMR spectroscopy.

**Figure 6:**
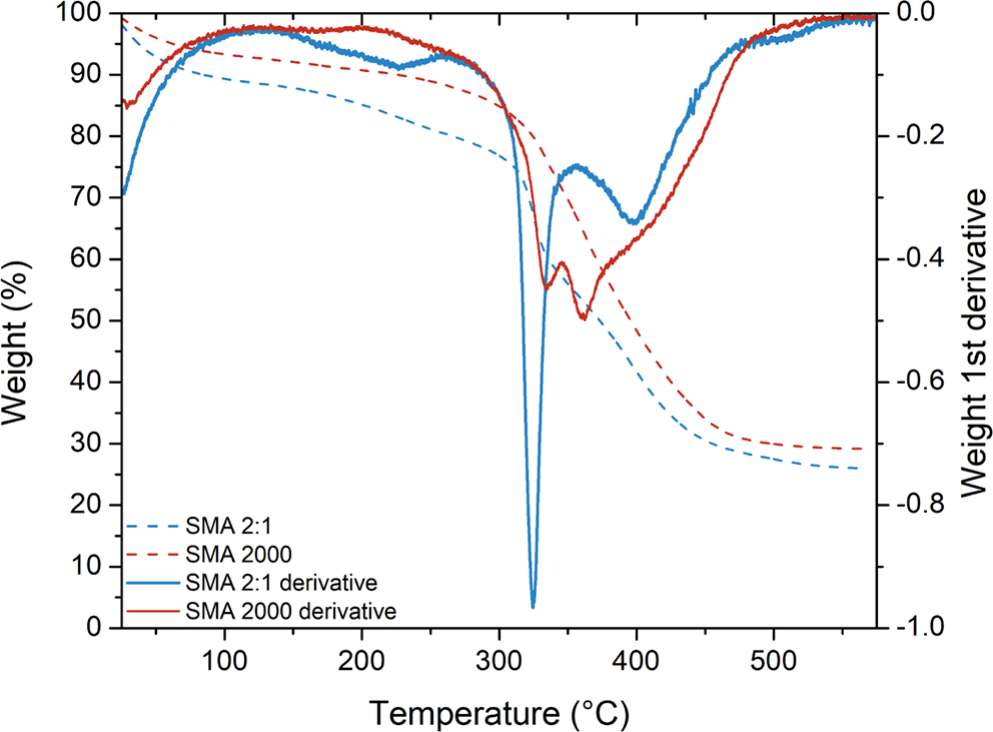
Thermogravimetric analysis of SMA 2:1 and SMA2000. Dotted lines indicate the weight (%) as a function of temperature (°C). The solid lines are indicative of the derivative of the weight as a function of temperature (°C).

SMA 2:1, SMA2000, and two slightly more hydrophilic copolymers (SMA 1:1 and SMA 1.5:1) were investigated further to determine the effect of varying copolymer composition and microstructure on the solution behavior of the copolymers. This was assessed via titration of the copolymers with divalent cations (Mg^2+^/Ca^2+^) or HCl. Furthermore, the copolymers were used to mediate the solubilization of DMPC/POPC vesicles, as the successful interaction of SMA copolymers with lipid bilayers is strongly dependent on the application of the appropriate amphiphilicity and charge along the backbone.

The protonation or chelation of divalent cations to the SMA carboxylate functional groups screens anionic charges, thereby making interactions between the copolymer and the aqueous medium increasingly unfavorable. A significant decrease in charge along the SMA backbone facilitates precipitation of the copolymer, which can be monitored via turbidimetry (Figure 7). The titration of selective SMA copolymers with HCl (Figure 7A) indicated that all copolymers retain enough charge density along the backbone to remain soluble in water between pH 4 and 12, with the exception of SMA 2:1, which exhibited an increase in optical density at slightly higher pH (ca. 5). The ^13^C NMR spectroscopic analysis of SMA 2:1 suggests this copolymer constitutes the highest SSS triad fraction; therefore, protonation of the MA units would facilitate the formation of hydrophobic domains at higher comparative pH values. All copolymers exhibited similar tolerance to Mg^2+^ as they maintained their aqueous solubility up to 15 mM, whereas 1:1 SMA exhibited the greatest tolerance to Mg^2+^ (20 mM) due to its higher MA content. A larger variation in solution behavior with varying copolymer compositions was observed for Ca^2+^ titrations, as increasing S:MA (SMA 1:1 < SMA 1.5:1 < SMA 2:1 < SMA2000) generally decreased Ca^2+^ tolerance from approximately 20 mM to 10 mM. To critically examine the effect of decreasing charge density of SMA chains with varying comonomer composition, *Z* ratios were calculated for each [M^2+^] (defined in Figure 8). While the precipitation of SMA occurs over a narrow range of [M^2+^], the inflection point of the resulting curves (Figure S3) was defined as the ratio of cationic/anionic charges required for loss of aqueous solubility to facilitate comparison between copolymers. These Z ratios were subsequently plotted as a function of the S/MA ratio in Figure 8.

**Figure 7:**
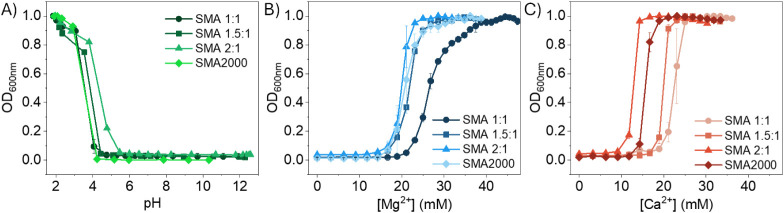
Titration curves for SMA 1:1, SMA 1.5:1, SMA 2:1, and SMA2000 as a function of optical density (at 600 nm). Polymer solutions at 10 mg·mL^–1^ (in DI water for acid titrations or Tris HCl buffer, 50 mM, for M^2+^ titrations) were titrated with HCl (0.5 M), Mg^2+^ (0.1 M) or Ca^2+^ (0.1 M) until the copolymers precipitated.

**Figure 8:**
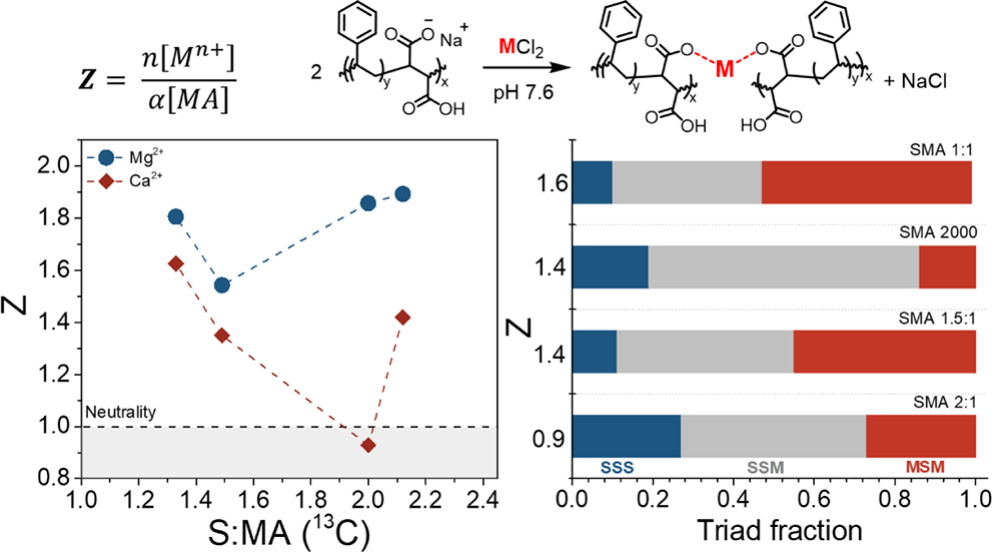
*Z* ratios obtained from the titration of SMA 1:1, SMA 1.5:1, SMA 2:1, and SMA2000 (left) with Mg^2+^/Ca^2+^ plotted as a function of the S:MA ratio (determined via ^13^C NMR spectroscopy). All M^2+^ titrations were performed at pH 7.6, and therefore α = 1. Dashed lines are included solely to guide the eye. Triad fractions (Table S2) determined for each SMA copolymer are presented for each Ca^2+^ Z ratio (right).

All SMA copolymers assessed required Mg^2+^ in stoichiometric excess compared to carboxylate groups and the Mg^2+^ Z ratio required for precipitation of the copolymer from solution was generally independent of the copolymer composition (Figure 8). For all samples, the Ca^2+^ Z ratio was lower than the corresponding Mg^2+^ Z ratio, and additionally, the Ca^2+^ Z ratio was somewhat dependent on the S:MA composition, whereby increasing S content resulted in lower Z. The disparate ionic radii and coordination behavior of Ca^2+^ and Mg^2+^ are likely to play a role in the lower Z ratios required for Ca^2+^-mediated precipitation of SMA. Ca^2+^ has a larger ionic radius (0.99 Å) than Mg^2+^ (0.65 Å) and generally prefers higher coordination numbers than Mg^2+^, properties that have been exploited to investigate the destabilization of SMALPs using various divalent cations with increasing ionic radii.^55,56^ SMA 2:1 had a Ca^2+^ Z ratio of 0.9, significantly lower than the Z ratio determined for SMA2000 (1.4) despite the two copolymers having similar S:MA composition (Figure 8). SMA 2:1 and SMA2000 are, however, likely to have different microstructures (and conformation in solution), as their triad distribution is considerably dissimilar (Figure 8). SMA 2:1 has the highest fraction of the SSS triad, which yields hydrophobic domains along the backbone, promoting a collapsed coil conformation, and therefore, comparatively less Ca^2+^ is required to facilitate precipitation.

The influence of SMA composition and microstructure on its behavior at a lipid–water interface was investigated via light scattering analyses. Vesicles constituting a bilayer of synthetic lipids, such as saturated 1,2-dimyristoyl-*sn*-glycero-3-phosphocholine (DMPC) or monounsaturated 1-palmitoyl-2-oleoyl-glycero-3-phosphocholine (POPC), were prepared and treated with SMA of varying S:MA composition. The solubilization of POPC vesicles was monitored via turbidimetry for up to 1.1 h at 25 °C, where a decrease in optical density corresponds to the formation of smaller lipid particles (SMALPs), which scatter light to a lesser extent than the corresponding lipid vesicle (Figure 9). A minor drop in optical density was observed for SMA 1:1 and SMA 1.5:1, where DLS analysis of the resulting lipid particles indicated that the hydrodynamic diameter had decreased slightly (560–880 nm) compared to the initial POPC vesicles (1230 nm). Solubilizations conducted using SMA 2:1 and SMA2000, however, did result in a significant decrease in optical density, resulting in the formation of lipid particles with hydrodynamic diameters characteristic of SMALPs (7–8 nm).^9^ Conversely, the exposure of DMPC vesicles to the same SMA copolymers and experimental conditions resulted in successful solubilization in all cases, as the optical density of all samples dropped to approximately zero in under 20 s with the resulting SMALPs, exhibiting hydrodynamic diameters of 6–9 nm (Figure S5). Therefore, the difference in overall S:MA composition and microstructure did not have a significant influence on the interaction between SMA and a lipid bilayer constituting saturated acyl chains, but these properties appeared to have a considerable influence on interactions with a lipid bilayer constituting monounsaturated acyl chains. A recent study by Lund et al. investigated the formation of DMPC/POPC SMALPs (using SMA 3:1) via small-angle X-ray scattering analyses.^57^ Their findings suggested that well-defined POPC vesicles have the capacity for multilamellarity (up to 4–5 monolayers), as opposed to the unilamellarity of well-defined DMPC vesicles, and additionally suggested that nonsaturated lipids such as POPC may be comparatively more resistant to bilayer disruption derived from packing defects introduced via styrene insertion.^57^ It is plausible that SMA 1:1 (55% S) and SMA 1.5:1 (60% S) have insufficient styrene content to successfully induce bilayer disruption compared to SMA 2:1 (67% S) and SMA2000 (68% S), yielding an unsuccessful solubilization (Figure 9). Despite the comparable styrene contents of SMA 2:1 and SMA2000, the solubilization of POPC vesicles using the former is considerably slower than the latter. SMA 2:1 has higher SSS (0.27) and MSM (0.27) triad fractions compared to SMA2000 (0.19 and 0.14, respectively), which constitutes predominantly SSM triads (0.67). Thus, the microstructure and the resulting conformation of the copolymer at the lipid–water interface appear to be dominating factors for the disparate solubilization kinetics observed for SMA 2:1 and SMA2000.

**Figure 9:**
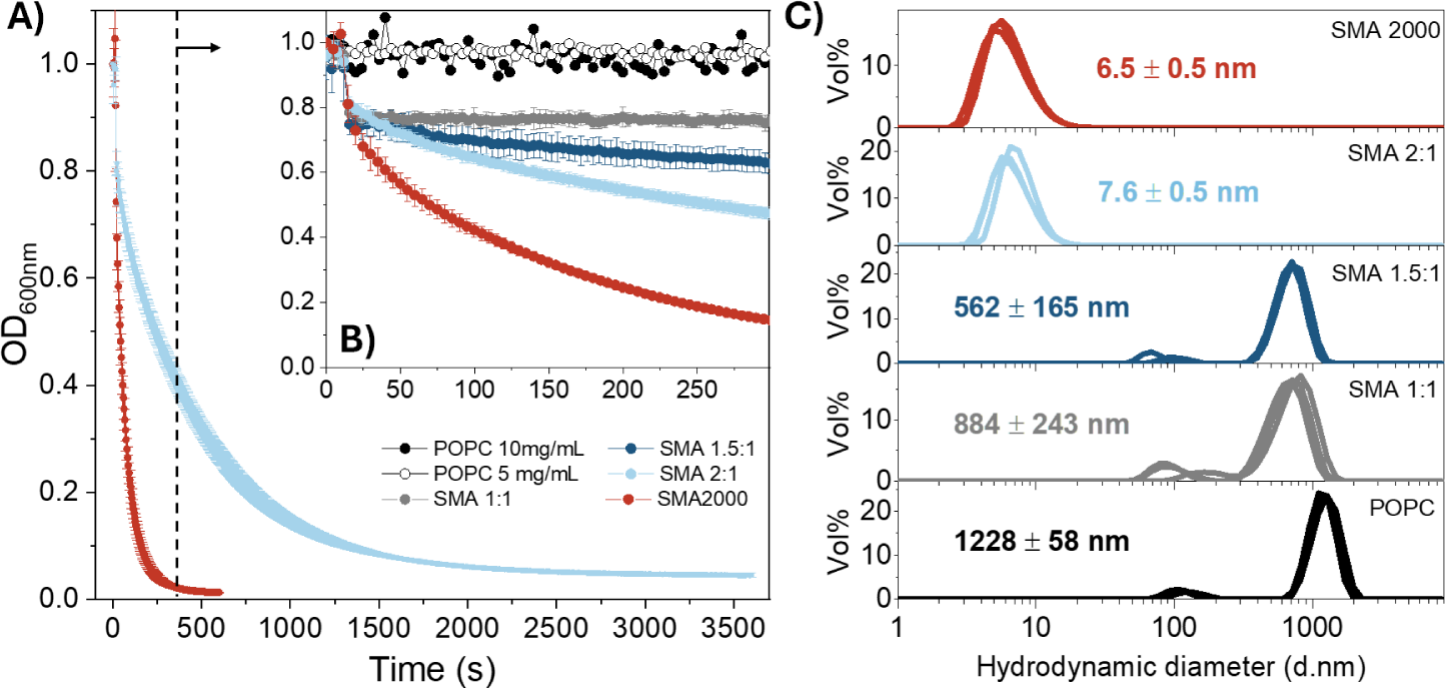
(A) Turbidimetric analysis for the solubilization of POPC vesicles (5 mg/mL in Tris HCl buffer, 50 mM, pH 7.6) using SMA copolymers (25 mg/mL in Tris HCl buffer) with varying S/MA composition at 25 °C for 1.1 h. (B) Similar turbidimetric analyses to (A) but conducted over 5 min. (C) DLS analysis of particles synthesized during solubilization experiments, with samples measured in triplicate.

Historically, the overall chemical composition and amphiphilicity for SMA-type copolymers are considered important, contributing factors for the successful solubilization of lipid membranes. Therefore, many studies attempt to emulate the amphiphilicity of the industry gold standard SMA2000 via postpolymerization modification of MAnh units (along the parent SMAnh backbone) or via copolymerization of alternative comonomer analogues.^4,7^ Partial or quantitative postpolymerization modification of SMAnh has been effectively employed to vary SMA amphiphilicity. For partially modified SMA, the distribution of modified units along the copolymer backbone is probability-driven.^7^ Alternatively, quantitative modification of MA units along the SMA backbone significantly reduces the overall charge density, which increases the sensitivity of the copolymer to divalent cations and low pH.^6^ The copolymerization of S or MAnh analogues often yields copolymers with strong alternating character, thereby limiting the opportunity for variable microstructure, requiring that the comonomers are carefully selected to yield desirable properties.^4^ In this study, we have demonstrated that SMA copolymers can be synthesized with low *Đ* and homogeneous comonomer distribution among different copolymer chains due to the application of the RAFT polymerization technique. Additionally, the copolymerization of S and MA (with reduced electron-acceptor character compared to MAnh) affords a facile approach for producing nonalternating SMA with tunable S:MA composition without the need for pre- or postmodification of comonomer units. This work therefore presents an alternative synthetic approach toward the synthesis of SMA with a similar composition to the widely used SMA2000, which presents exciting future prospects for the field of membrane protein-related research.

## Conclusion

SMA was synthesized via the direct RAFT-mediated copolymerization of styrene and maleic acid to afford a copolymer that has the same overall copolymer composition but a different monomer sequence distribution (microstructure) to SMA derived from the hydrolysis of SMAnh with the same overall chemical composition. The S-MA copolymerization was shown to obey the restricted terminal model in comparison to the S-MAnh system, which is known to be best described by the restricted penultimate model. The deviation in the kinetic model description is most likely a result of the differing monomer electronics, where the alkene in MA is less electron-deficient than that in MAnh. SMA 2:1 afforded via the copolymerization of S and MA was found to have dissimilar properties (solid-state thermal stability, solution pH tolerance, and cation tolerance) to commercially available equivalent SMA 2:1 copolymer (SMA2000, afforded through the copolymerization of S and MAnh). It was further demonstrated that the two SMA copolymers of comparable amphiphilicity (i.e., similar S and MA composition) behaved dissimilarly during the solubilization of synthetic lipid vesicles. This was hypothesized to be a result of the different microstructure of the copolymers, as a result of varying copolymer triad distributions (i.e., the composition of SSS, SSM and MSM fractions). This work therefore highlights that copolymer microstructure plays a significant role during the formation of SMALPs, in addition to the overall amphiphilicity and charge density of the SMA copolymer.

## Supplementary Material



## Notes

Additional experimental details, methods and results (PDF)

## †

Leibniz-Institut für Polymerforschung Dresden e.V., Hohe Straße 6, D-01069 Dresden, Germany

## Abbreviations

SMA: poly­(styrene-*co*-maleic acid)
SMAnh: poly­(styrene-*co*-maleic anhydride)
SMALP: poly­(styrene-*co*-maleic acid) lipid particle
RAFT: reversible addition–fragmentation chain-transfer
S: styrene
MAnh: maleic anhydride
CSTR: continuous stirred tank reactor
MA: maleic acid
DMPC: 1,2-dimyristoyl-*sn*-glycero-3-phosphocholine
POPC: 1-palmitoyl-2-oleoyl-glycero-3-phosphocholine
NMR: nuclear magnetic resonance spectroscopy
SEC: size exclusion chromatography
TGA: thermogravimetric analysis
DLS: dynamic light scattering
CTA: chain transfer agent
TTC: trithiocarbonate
DTC: dithiocarbamate
DTB: dithiobenzoate
AIBN: azobis­(isobutyronitrile)
