# Supplementary material for: The RAFT-Mediated Synthesis of Poly(styrene-co-maleic acid) through Direct Copolymerization of Maleic Acid
Source: Macromolecules. 2025 Jul 31;58(15):8409–18. doi: 10.1021/acs.macromol.5c01372 (PMC12356055; doi:10.1021/acs.macromol.5c01372)
Supplement: Supplementary file 1 [file ma5c01372_si_001.pdf]

The RAFT-mediated synthesis of poly(styrene-*co*-maleic acid) through direct copolymerization of maleic acid

*Michael-Phillip Smith, Lauren E. Ball, Bert Klumperman**

Department of Chemistry and Polymer Science, University of Stellenbosch, Private Bag X1, Matieland 7602, South Africa.

Contents

Experimental data 1
- Triad distribution calculations 1
- SMA copolymerization kinetic calculations 1
- S:MA determination methods 1
  - Method 1 1
  - Method 2 1
  - Method 3 2
- Copolymerization composition kinetics data 4
- SMA library for testing data 4

Computational data 8
- Calculated energies 8
- Atom coordinates 6

References 18

Experimental data

Triad distribution calculations

Triads were calculated according to the method stipulated in the literature,[1] according to the ^{13}C NMR spectroscopy ppm ranges noted by Ha.[2]

SMA copolymerization kinetic calculations

S and MA conversion was calculated utilizing the vinylic protons integrals determined *via* ^{1}H NMR spectroscopy. DMF was utilized as an internal standard with the integral ranges of the monomers stipulated below.

S = 5.760 – 5.570 ppm; MA = 6.340 – 6.160 ppm; DMF internal standard = 7.920 – 7.820 ppm

Equations S1 and S2 were used to calculate 1) monomer conversion and 2) M_n^{theo}.

Equation S1:

$$a_S = \frac{(I_{t0}^{st} - I_{tx}^{st})}{I_{t0}^{st}} \quad \& \quad a_{MA} = \frac{(I_{t0}^{MA} - I_{tx}^{MA})}{I_{t0}^{MA}}$$

Equation S2:

$$M_n^{theo} = \frac{([S] \times MW_S \times a_S)}{[CTA]} + \frac{([MA] \times MW_{MA} \times a_{MA})}{[CTA]} + MW_{CTA}$$

S:MA determination methods

S:MA was determined and supported via three methods of determination. All methods produced very similar S:MA amounts.

Method 1

S:MA was determined *via* ^{1}H NMR spectroscopy conversion. Where the signals indicated prior in the SI were utilized to determine S and MA consumed in the copolymerization. This method of determination relies on the assumption that all monomer consumed is incorporated into the growing polymer chain. Which as a results of this method being similar to other methods, would likely be a good assumption.

Method 2

S:MA was determined *via* ^{13}C NMR spectroscopy. Where a ratio is taken between the S and MA carbon backbone signals.

Method 3

S:MA was determined *via* triad composition and calculated *via* the equation stipulated in the work by Barron and coworkers.[1]

$$\frac{F_S}{F_{MA}} = 1 + \frac{2A_{SSS} + A_{SSM+MSS}}{2A_{MSM} + A_{SSM+MSS}}$$

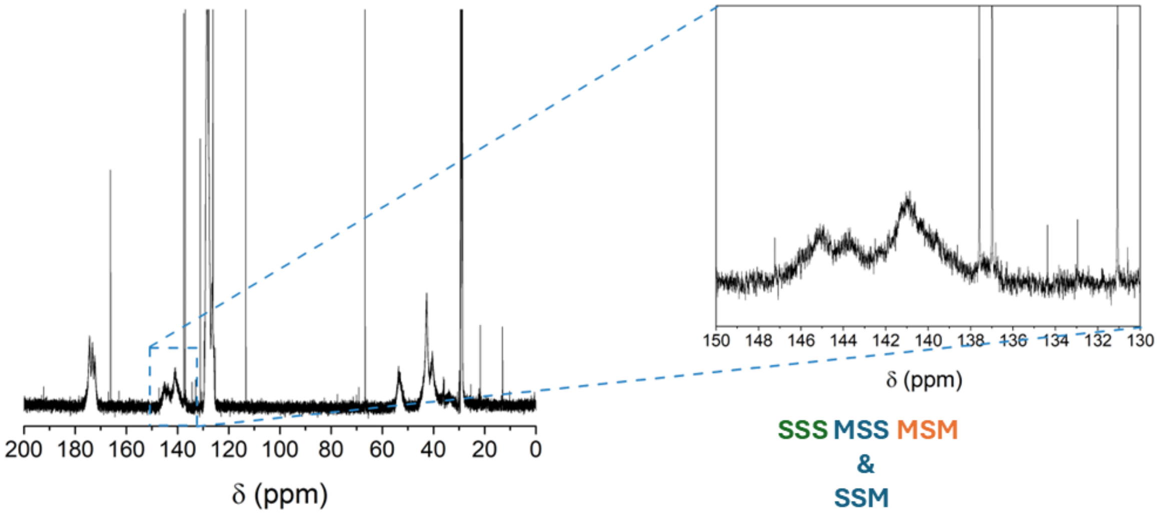

Figure S1. Representative quantitative ^{13}C NMR spectrum of poly(styrene-*co*-maleic acid) utilized for triad destribution determination. NMR spectrum was aquired in acetone-d_6.

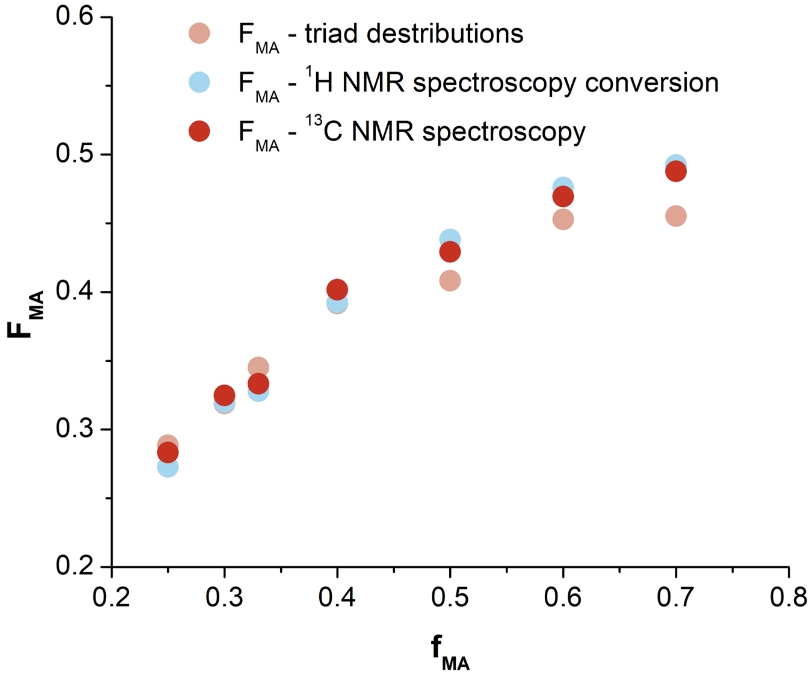

Figure S2. Comparison of the methods of F_{MA} determination.

Copolymerization composition kinetics data

Table S1. Data on copolymers synthesized for the determination of the reactivity ratios of the SMA system.

f_{MA}	α_S (30 h)	α_{MA} (30 h)	SSS	SSM + MSS	MSM	F_{MA} (^{1}H NMR conv %)	F_{MA} (^{13}C NMR)
0.25	0.32	0.36	0.35	0.49	0.16	0.27	2.53
0.30	0.31	0.34	0.28	0.51	0.22	0.32	2.08
0.33	0.40	0.39	0.23	0.48	0.29	0.33	2.00
0.40	0.31	0.30	0.14	0.44	0.42	0.39	1.49
0.50	0.50	0.39	0.10	0.41	0.48	0.44	1.33
0.60	0.71	0.43	0.02	0.30	0.68	0.48	1.13
0.70	0.77	0.32	0.04	0.24	0.71	0.49	1.05

SMA library for testing data

Table S2. Copolymers utilized for further analysis in lipid nanodisc applications. $ = determined with comonomer polymerization conversion. * = based on triad distributions.

Sample code	f_{MA}	α_S (30 h)	α_{MA} (30 h)	F_{MA}^*	$F_{MA}^\$$	SSS	SSM + MSS	MSM	M_n^{theo} (g·mol^{-1})	M_n^{SEC} (g·mol^{-1})	*Đ*
SMA 1:1	0.50	0.39	0.31	0.41	0.45	0.10	0.37	0.52	7910	5290	1.33
SMA 1.5:1	0.40	0.43	0.40	0.40	0.40	0.11	0.44	0.45	7780	5090	1.20
SMA 2:1	0.33	0.35	0.34	0.33	0.33	0.27	0.46	0.27	7710	4730	1.30
SMA2000 hydrolyzed	-	-	-	0.32	-	0.19	0.67	0.14	-	4200	1.90
SMA2000	-	-	-	0.32	-	0.19	0.67	0.14	-	3560	1.89

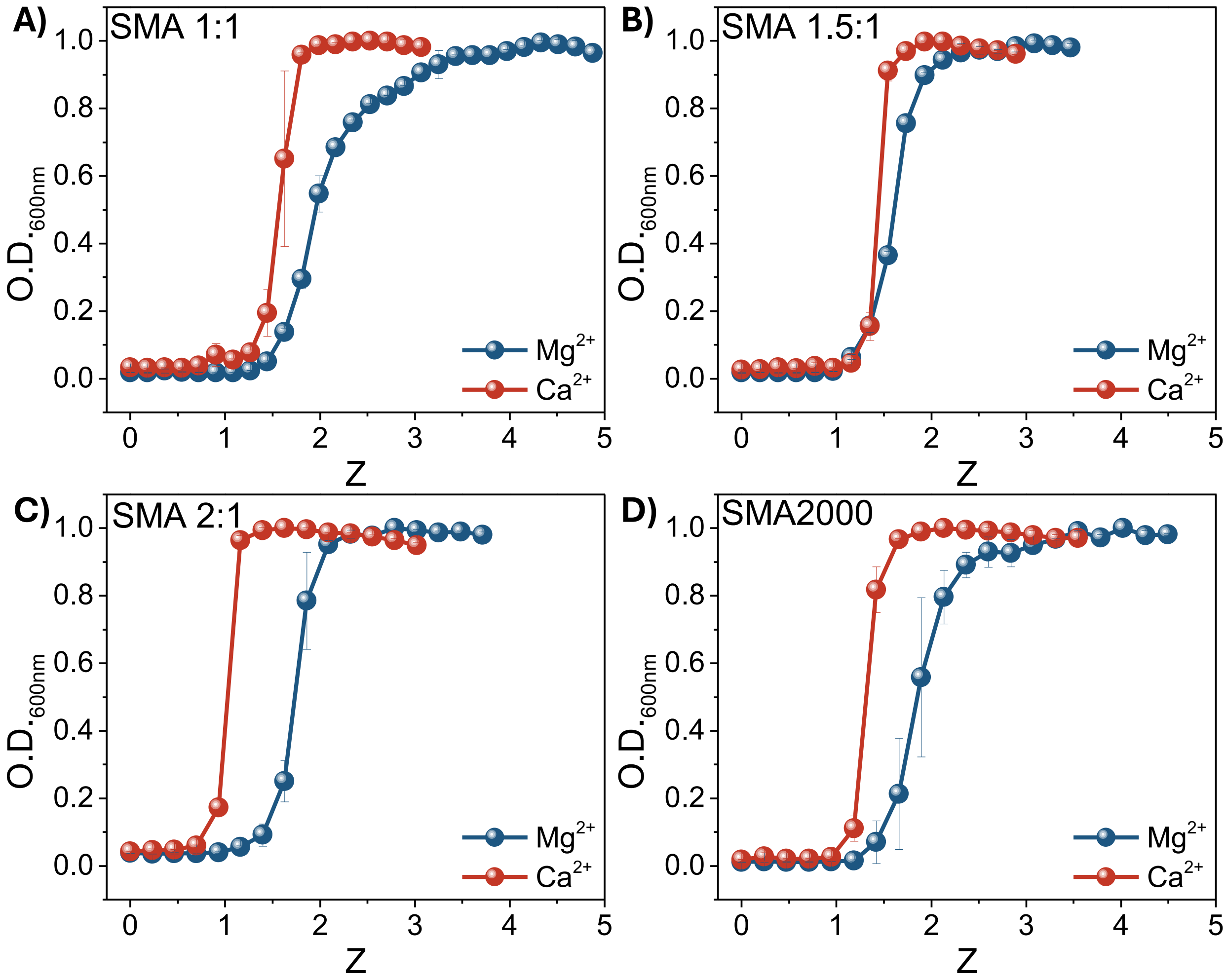

Figure S3. Optical density (at 600 nm) of Mg^{2+}/Ca^{2+} titrated SMA copolymer solutions as a function of the charge ratio, Z. Z ratios were calculated using the **Equation 3**.

Equation 3. Charge ratio between divalent cations and MA anionic carboxylate functional groups. [M^{n+}] can be calculated as the cumulative amount of Mg^{2+}/Ca^{2+} added during the titration is known. [MA] is calculated according to the MA composition determined *via* ^{13}C NMR spectroscopic analysis (values listed in Table S2). The values n and α represent the valency of the cation and the charge per MA unit respectively, where MA has a charge of -1 at the pH assessed (7.6).

$$\boldsymbol{Z} = \frac{n[M^{n+}]}{\alpha[MA]}$$

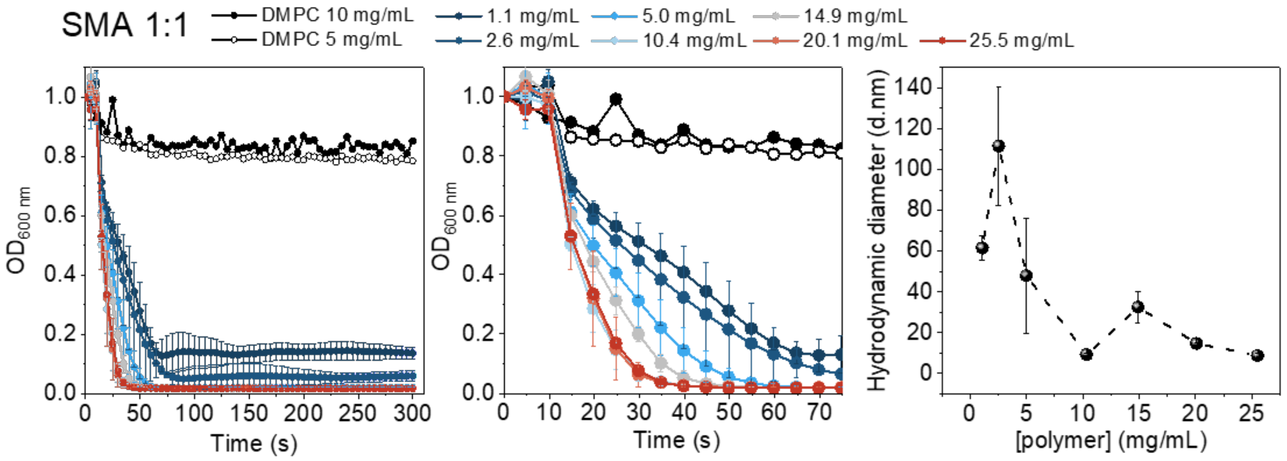

Figure S4. Dose response curves for the solubilization of DMPC vesicles using SMA 1:1. The optical density (at 600 nm) of DMPC vesicles (10 mg/mL in Tris HCl buffer, 50 mM, pH 7.6) at 25 °C was recorded for 5 min (left); the solution diluted to 5 mg/mL with Tris HCl buffer and the optical density recorded for 5 min. These control samples indicate that the optical density of the solution is not affected by dilution of the sample once the copolymer solutions are added in subsequent solubilization experiments, but rather due to interactions of the copolymer with the lipid bilayer. SMA solutions at varying concentration were prepared to assess the effect of the lipid:polymer ratio on SMALP formation. Lipid:polymer ratios between 5:1 and 1:5 were assessed as indicated in the plot on the left, with the plot in the middle honing in on the first ~75 seconds of solubilization. Overall, the highest lipid:polymer ratio of 1:5 was selected for subsequent solubilization experiments as it yielded fast solubilization kinetics and the smallest SMALPs (8.8 ± 1.3 nm), determined via DLS (right).

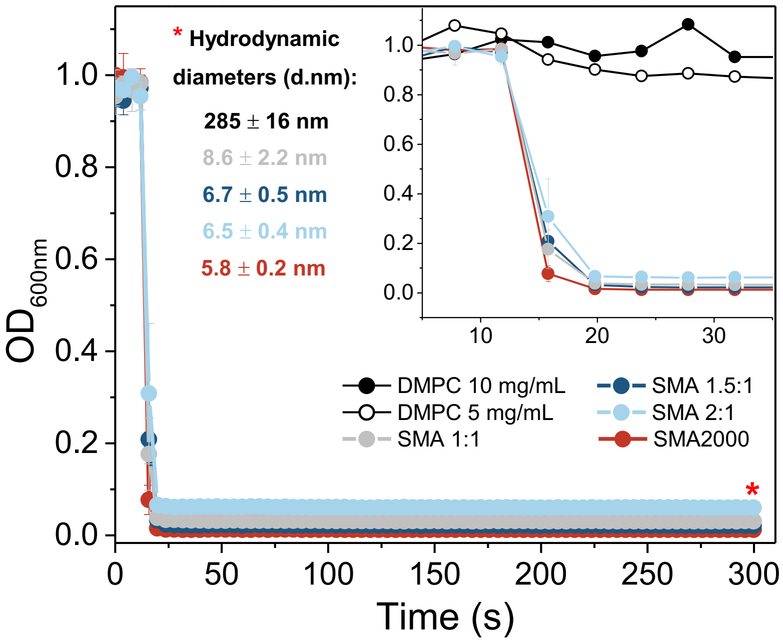

Figure S5. Turbidimetric analysis of DMPC vesicle solubilizations using SMA copolymers with varying S:MA composition. The lipid:polymer ratio employed was 1:5, with solubilizations conducted at 25 °C for 5 min. Subsequently, the synthesized lipid particles were characterized using DLS, with the hydrodynamic diameters listed in the plot (colour coded according to sample). The inset data represents the first ~25 seconds of each solubilization to elucidate slight differences in the solubilization rate of the copolymers. All copolymers assessed solubilized DMPC vesicles efficiently due to the rapid decrease in optical density and the formation of lipid particles ranging between ~6–9 nm, considerably smaller than the initial DMPC vesicles (~286 nm).

Computational data

Calculated energies

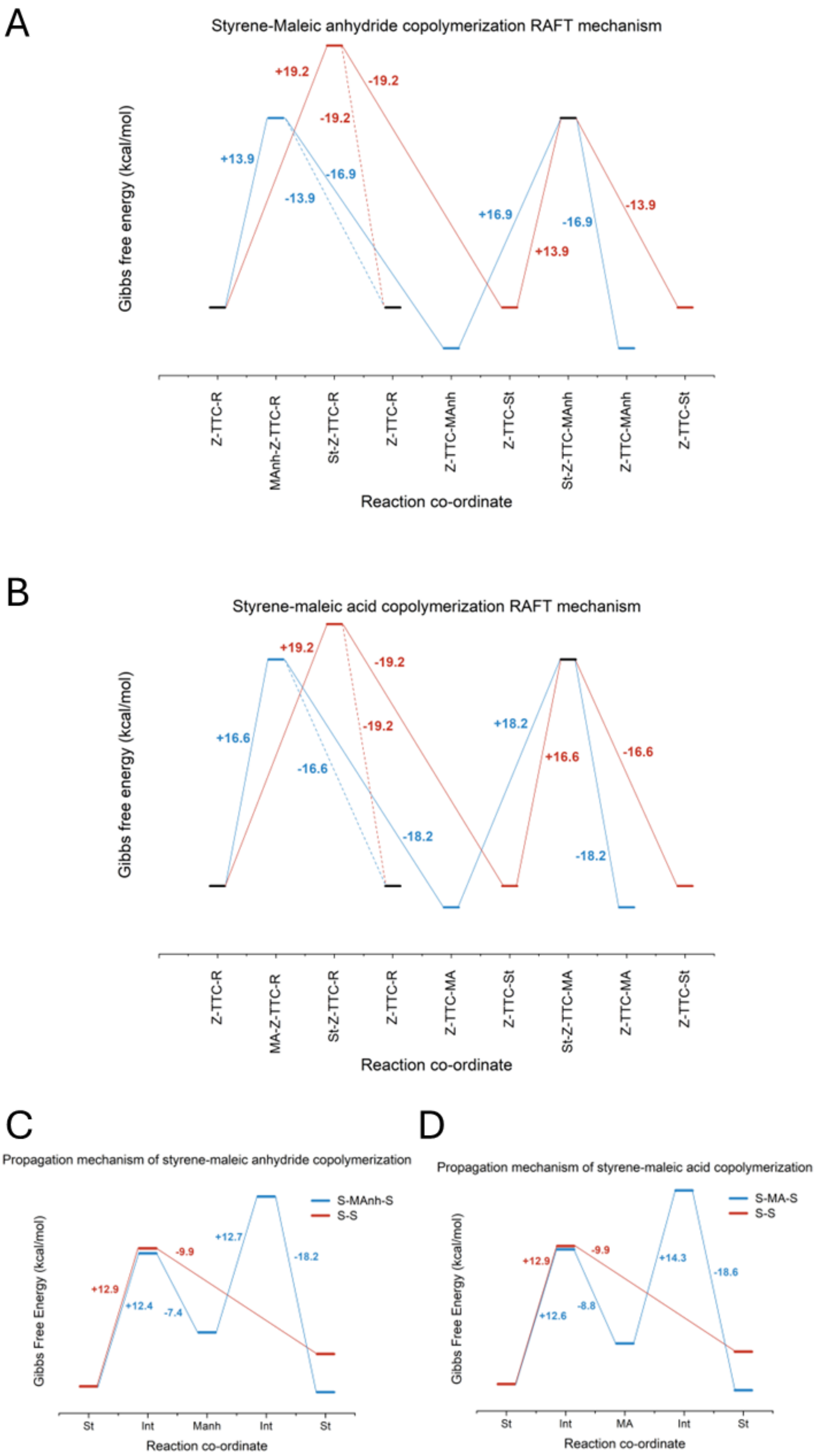

Figure S6. Computational analysis of the copolymerization of SMAnh and SMA. Gibbs free energies ($kcal{\cdot}mol^{-1}$) were calculated for the interaction of the TTC with A) MAnh and B) MA. The energy of a propagating radical reacting with monomer was modelled for C) MAnh and D) MA.

Equations for the Terminal Model (TM):

$$q = \frac{f_S}{f_M} \quad \text{(S3)}$$

$$p_{SM} = \frac{1}{1 + r_S q} \quad \text{(S4)}$$

$$p_{MS} = \frac{1}{1 + \frac{r_M}{q}} \quad \text{(S5)}$$

$$\frac{F_S}{F_M} = \frac{p_{MS}}{p_{SM}} \quad \text{(S6)}$$

$$F_{SSS} = (1 - p_{SM})^2 \quad \text{(S7)}$$

$$F_{SSM+MSS} = 2p_{SM}(1 - p_{SM}) \quad \text{(S8)}$$

$$F_{MSM} = p_{SM}^2 \quad \text{(S9)}$$

Equations for the restricted Penultimate Model (rPUM):

$$p_{SSM} = \frac{1}{1 + r_{SS} q} \quad \text{(S10)}$$

$$p_{MSS} = \frac{r_{MS} q}{1 + r_{MS} q} \quad \text{(S11)}$$

$$\frac{F_S}{F_M} = 1 + \frac{p_{MSS}}{p_{SSM}} \quad \text{(S12)}$$

$$F_{SSS} = \frac{p_{MSS}(1 - p_{SSM})}{p_{SSM} + p_{MSS}} \quad \text{(S13)}$$

$$F_{SSM+MSS} = \frac{2p_{SSM} p_{MSS}}{p_{SSM} + p_{MSS}} \quad \text{(S14)}$$

$$F_{MSM} = \frac{p_{SSM}(1 - p_{MSS})}{p_{SSM} + p_{MSS}} \quad \text{(S15)}$$

Atom coordinates

Z-TTC-R

Final energy = -1700.816424 hartree

S	0.21960	0.71150	0.13570
C	1.95260	0.61050	-0.18030
S	2.46180	-1.06060	0.12100
S	2.91920	1.85630	-0.68980
C	-0.25560	2.38360	-0.45940
C	-1.76810	2.56550	-0.28430
C	-2.23470	3.98900	-0.64270
C	-2.05360	4.36940	-2.11790
C	4.31860	-0.99040	0.28170
C	4.76540	-0.41940	1.61400
C	4.82500	-2.42100	0.03600
C	4.35720	-0.98410	2.83380
C	4.81990	-0.47040	4.04670
C	5.70170	0.61660	4.06130
C	6.11630	1.18340	2.85380
C	5.64850	0.66970	1.63940
H	0.30270	3.11910	0.12630
H	0.05260	2.46680	-1.50490
H	-2.30390	1.83630	-0.90740
H	-2.04200	2.35790	0.75780
H	-3.29790	4.06750	-0.38060
H	-1.70520	4.71190	-0.00680
H	-2.47240	5.36320	-2.31530
H	-0.99690	4.39490	-2.41090
H	-2.56600	3.65410	-2.77550
H	4.65970	-0.33980	-0.52720
H	5.91910	-2.42270	0.09800
H	4.44490	-3.12440	0.78430
H	4.53430	-2.77680	-0.95840
H	3.66740	-1.82470	2.83820
H	4.49120	-0.91860	4.98070
H	6.05960	1.01720	5.00560
H	6.79960	2.02880	2.85220
H	5.97110	1.11950	0.70350

Z-TTC-MAnh

Final energy = -1770.461733 hartree

S	0.53180	0.88080	-0.65730
C	2.13200	0.77370	0.06070
S	2.72160	-0.89810	-0.17890
S	2.94890	1.99110	0.82450
C	0.01300	2.61660	-0.34150
C	-1.38510	2.83770	-0.92710
C	-1.86480	4.27900	-0.69140
C	-3.26420	4.53350	-1.26360
C	4.39910	-0.83290	0.54650
C	5.55450	-0.49820	-0.40440
C	4.77340	-2.18830	1.13790
O	6.05990	-2.51300	0.74820

C	6.58670	-1.56760	-0.13100
O	4.14050	-2.90920	1.85890
O	7.70200	-1.68820	-0.55640
H	0.02620	2.77990	0.73940
H	0.75170	3.27650	-0.80450
H	-1.37620	2.62860	-2.00490
H	-2.09650	2.13780	-0.46910
H	-1.86500	4.48850	0.38710
H	-1.14920	4.97770	-1.14600
H	-3.58170	5.56690	-1.08170
H	-3.28620	4.36170	-2.34710
H	-4.00660	3.86880	-0.80370
H	4.34610	-0.11480	1.37490
H	5.27110	-0.55430	-1.46080
H	5.98490	0.49040	-0.22580

Z-TTC-MA

Final energy = -1846.903265 hartree

S	0.40750	1.09600	-0.05720
C	2.16470	1.17930	0.02190
S	2.75750	-0.33160	0.74890
S	3.11010	2.43690	-0.49050
C	-0.08050	2.68850	-0.83520
C	-1.60800	2.75290	-0.93580
C	-2.06850	4.06400	-1.59260
C	-3.59500	4.16500	-1.69550
C	4.57630	-0.12270	0.73770
C	5.21360	-1.50870	0.99320
C	5.04990	0.88560	1.78830
C	6.61480	-1.61770	0.41850
O	4.43790	1.23390	2.77580
O	6.99740	-1.08200	-0.60390
O	6.29750	1.30210	1.50350
O	7.38540	-2.44210	1.15410
H	0.31550	3.50000	-0.21760
H	0.38950	2.73980	-1.82130
H	-1.98090	1.90250	-1.52170
H	-2.05060	2.67230	0.06600
H	-1.68180	4.91470	-1.01340
H	-1.62600	4.14160	-2.59540
H	-3.89600	5.10670	-2.16980
H	-4.00700	3.34190	-2.29280
H	-4.06190	4.12560	-0.70310
H	4.87160	0.23190	-0.25230
H	5.21860	-1.76600	2.05520
H	4.62510	-2.28020	0.48000
H	6.59490	1.90820	2.21190
H	8.25390	-2.53340	0.71310

St-Z-TTC-R

Final energy = -2011.069593 hartree

S	0.27730	1.78690	-1.40280
C	1.93940	1.39850	-0.94640
S	2.23050	0.76480	0.67780

S	3.19180	1.71150	-2.12850
C	-0.49730	2.42760	0.15950
C	-1.78920	3.19470	-0.14040
C	-2.93370	2.34890	-0.71580
C	-4.22760	3.15500	-0.88060
C	3.92510	-0.03120	0.61670
C	4.35150	-0.21070	2.06180
C	3.92830	-1.31540	-0.21640
C	3.82620	-1.23540	2.86590
C	4.22750	-1.37270	4.19800
C	5.16090	-0.48820	4.74800
C	5.68890	0.53720	3.95700
C	5.28350	0.67470	2.62650
C	3.34400	3.60290	-2.19970
C	4.28050	3.90550	-3.37530
C	3.77490	4.19250	-0.87810
C	2.85730	4.93290	-0.11670
C	3.22610	5.49360	1.11120
C	4.52400	5.32160	1.59830
C	5.44910	4.58540	0.84750
C	5.07780	4.02520	-0.37630
H	-0.69660	1.58470	0.82870
H	0.23570	3.08620	0.63360
H	-2.11810	3.63690	0.81130
H	-1.57050	4.03470	-0.81390
H	-2.63850	1.93290	-1.68850
H	-3.11540	1.49120	-0.05270
H	-5.03280	2.53610	-1.29380
H	-4.57080	3.55150	0.08370
H	-4.07930	4.00660	-1.55690
H	4.57620	0.70890	0.14520
H	4.94040	-1.73930	-0.22410
H	3.24240	-2.06730	0.18530
H	3.63930	-1.10770	-1.25100
H	3.09890	-1.93130	2.45630
H	3.81030	-2.17190	4.80550
H	5.47370	-0.59740	5.78340
H	6.41510	1.23020	4.37430
H	5.69410	1.47760	2.01850
H	2.33710	3.95560	-2.43860
H	4.37780	4.99100	-3.49320
H	5.28310	3.49210	-3.22070
H	3.88020	3.49320	-4.30770
H	1.84510	5.07320	-0.48950
H	2.49800	6.06270	1.68410
H	4.81390	5.75430	2.55250
H	6.46140	4.44500	1.21870
H	5.80570	3.44630	-0.93920

MAnh-Z-TTC-R

Final energy = -2080.726224 hartree

S	2.37420	1.68190	-1.24530
C	2.69810	-0.01990	-1.53950
S	4.34650	-0.62430	-1.42050

S	1.57400	-0.96860	-2.50490
C	0.70040	1.67390	-0.44450
C	0.32450	3.07790	0.03980
C	0.09930	4.11500	-1.06960
C	-0.37500	5.46530	-0.51920
C	4.95780	-0.08400	0.27130
C	4.15490	-0.67050	1.41250
C	6.44870	-0.45170	0.31160
C	4.10680	-2.05370	1.65490
C	3.39360	-2.56160	2.74260
C	2.71400	-1.69590	3.60800
C	2.75240	-0.31920	3.37480
C	3.46660	0.18710	2.28310
C	1.37940	-2.55410	-1.53150
C	0.90390	-3.69930	-2.43070
C	-0.52570	-3.94980	-2.00630
O	-0.81580	-3.19040	-0.88080
C	0.27450	-2.40960	-0.50440
O	-1.35380	-4.67590	-2.48710
O	0.23420	-1.74600	0.49680
H	-0.02770	1.30230	-1.17170
H	0.75150	0.97200	0.39090
H	-0.60450	2.96690	0.61810
H	1.08390	3.44320	0.74460
H	1.02680	4.26100	-1.63920
H	-0.64320	3.72430	-1.78050
H	-0.52910	6.19090	-1.32650
H	-1.32410	5.35980	0.02270
H	0.36080	5.88910	0.17610
H	4.86000	1.00360	0.28880
H	6.87390	-0.11350	1.26280
H	6.60580	-1.53330	0.23670
H	6.99560	0.03520	-0.50250
H	4.62450	-2.73990	0.98880
H	3.36660	-3.63500	2.91550
H	2.15760	-2.09340	4.45330
H	2.22700	0.36270	4.03840
H	3.49270	1.25980	2.10760
H	2.33360	-2.74310	-1.03710
H	0.93930	-3.48340	-3.50300
H	1.48260	-4.61470	-2.26210

MA-Z-TTC-R

Final energy = -2157.169803 hartree

S	0.48400	0.93280	-1.04700
C	2.04830	0.43570	-0.41970
S	3.13230	1.51360	0.44110
S	2.44460	-1.25590	-0.71300
C	0.50340	2.77820	-1.01750
C	-0.74340	3.33330	-1.71720
C	-2.07420	3.03820	-1.01090
C	-3.26750	3.69150	-1.71810
C	3.36510	0.67860	2.14050
C	4.81720	0.31490	2.35760

C	2.77820	1.58870	3.22540
C	5.19880	-1.03440	2.40710
C	6.53400	-1.39610	2.61400
C	7.51180	-0.40990	2.77040
C	7.14330	0.93950	2.72300
C	5.80820	1.29800	2.52170
C	2.75760	-1.37510	-2.57610
C	2.55200	-2.83040	-2.99390
C	1.09030	-3.22190	-3.04930
C	4.16420	-0.90090	-2.85880
O	0.16900	-2.47330	-3.31730
O	5.14930	-1.61640	-2.91800
O	0.91840	-4.53860	-2.81430
O	4.21500	0.43550	-3.02710
H	0.55320	3.10830	0.02440
H	1.41050	3.10760	-1.53170
H	-0.60580	4.42220	-1.78030
H	-0.77970	2.96630	-2.75220
H	-2.23630	1.95330	-0.95560
H	-2.01740	3.39720	0.02680
H	-4.20650	3.47280	-1.19590
H	-3.15340	4.78240	-1.76120
H	-3.36440	3.32510	-2.74840
H	2.77210	-0.23620	2.07400
H	2.90070	1.11360	4.20720
H	3.27990	2.56170	3.25650
H	1.71090	1.75830	3.05160
H	4.44540	-1.80890	2.28250
H	6.80780	-2.44760	2.65120
H	8.55060	-0.68880	2.92800
H	7.89650	1.71410	2.84350
H	5.54150	2.35150	2.48660
H	2.03020	-0.71940	-3.05710
H	2.94980	-2.97280	-4.00840
H	3.10390	-3.52140	-2.35080
H	-0.03130	-4.75110	-2.91340
H	5.14910	0.69790	-3.15110

Styrene

Final energy = -309.665058 hartree

C	-0.80230	1.11320	0.19840
C	0.02440	0.05430	0.21190
C	1.49820	0.06290	0.27800
C	2.26120	1.24520	0.35860
C	3.65350	1.19420	0.41690
C	4.32030	-0.03810	0.39680
C	3.57820	-1.21950	0.31830
C	2.18310	-1.16700	0.26000
H	-1.87860	0.97390	0.14680
H	-0.44940	2.14100	0.23660
H	-0.41860	-0.94120	0.16820
H	1.76600	2.21220	0.37680
H	4.22280	2.11840	0.47870
H	5.40580	-0.07360	0.44250

H	4.08300	-2.18220	0.30240
H	1.61250	-2.09150	0.19900

Maleic anhydride

Final energy = -379.318205 hartree

C	-5.57000	-6.68130	-0.04980
C	-6.40360	-6.88380	1.16340
O	-7.64240	-7.37760	0.75480
C	-7.62650	-7.49200	-0.63530
C	-6.29470	-7.04120	-1.11540
O	-8.58000	-7.89760	-1.24580
O	-6.15330	-6.68970	2.32360
H	-4.55720	-6.30310	0.00070
H	-6.02950	-7.03460	-2.16470

Maleic acid

Final energy = -455.761910 hartree

C	0.05140	-0.01180	0.02540
C	1.50880	-0.01430	0.31270
C	-0.11530	2.51420	-0.03090
C	-0.65830	1.11700	-0.10750
O	0.19160	3.15480	-1.01430
O	2.21620	0.98410	0.40580
O	-0.10310	2.99540	1.22950
O	1.98910	-1.26740	0.44670
H	-0.43240	-0.97720	-0.08330
H	-1.72050	1.06210	-0.33070

H	0.20690	3.91880	1.17700
H	2.94850	-1.21880	0.63480

Styrene radical

Final energy = -310.248492 hartree

C	0.77450	0.75110	-0.21920
C	0.82700	-0.58930	0.44060
C	1.85730	-1.55640	0.33470
C	1.73930	-2.79280	1.04570
C	2.72260	-3.76970	0.97170
C	3.87150	-3.56640	0.18840
C	4.01380	-2.36220	-0.51900
C	3.03590	-1.37600	-0.45330
H	0.81840	1.56080	0.52630
H	1.58670	0.91620	-0.93230
H	-0.17580	0.88470	-0.75600
H	-0.01530	-0.84280	1.08320
H	0.85380	-2.96140	1.65530
H	2.60070	-4.69820	1.52460
H	4.64080	-4.33170	0.13110
H	4.89990	-2.19500	-1.12710
H	3.17670	-0.45510	-1.01110

Maleic anhydride radical

Final energy = -379.897946 hartree

C	-5.49170	-6.64900	-0.02320

C	-6.39670	-6.87970	1.17680
O	-7.61310	-7.36870	0.74110
C	-7.62220	-7.49330	-0.66760
C	-6.34270	-7.06160	-1.16280
O	-8.61000	-7.90880	-1.23830
O	-6.17670	-6.69680	2.34550
H	-5.17770	-5.59600	-0.06400
H	-4.57100	-7.24180	0.07820
H	-6.08240	-7.05630	-2.21290

Maleic acid radical

Final energy = -456.345230 hartree

C	0.12210	0.10080	0.02050
C	-0.70420	1.38170	0.00170
C	2.32430	-0.19670	1.29680
C	1.58890	0.30640	0.15600
O	1.83220	-0.81200	2.24240
O	-1.77430	1.49900	-0.56330
O	3.65280	0.07910	1.22380
O	-0.13820	2.37250	0.71800
H	-0.25290	-0.50810	0.85250
H	-0.11180	-0.43260	-0.90920
H	2.13490	0.84290	-0.61350
H	4.08530	-0.28860	2.01840
H	-0.73000	3.15090	0.69760

Styrene-styrene radical intermediate

Final energy = -619.913872 hartree

C	1.45680	0.54440	0.03890
C	1.20720	-0.92670	0.16080
C	2.00290	-1.94440	-0.42500
C	1.67150	-3.31970	-0.22310
C	2.43470	-4.33650	-0.78170
C	3.55990	-4.03370	-1.56740
C	3.90760	-2.69060	-1.78050
C	3.15200	-1.66340	-1.22470
C	3.81560	0.01660	5.07740
C	4.13520	-1.12350	4.44280
C	3.44090	-2.42290	4.52100
C	2.25650	-2.62200	5.25860
C	1.64310	-3.87350	5.30110
C	2.19580	-4.95910	4.60890
C	3.36880	-4.77790	3.87150
C	3.98180	-3.52260	3.82800
H	2.27940	0.87590	0.69430
H	1.73320	0.84130	-0.98110
H	0.56870	1.11560	0.32730
H	0.37360	-1.24670	0.78320
H	0.80330	-3.56510	0.38500
H	2.15940	-5.37360	-0.60550
H	4.15550	-4.83080	-2.00430
H	4.77880	-2.44750	-2.38460
H	3.44400	-0.63210	-1.39980
H	4.41270	0.91340	4.93690

H	2.96610	0.10200	5.75100
H	5.00940	-1.11870	3.79080
H	1.80760	-1.79420	5.80020
H	0.72880	-4.00400	5.87490
H	1.71440	-5.93310	4.64410
H	3.80700	-5.61200	3.32890
H	4.89310	-3.38940	3.24860

Styrene-maleic anhydride radical intermediate

Final energy = -689.563721 hartree

C	-7.49000	-7.21080	-0.04700
C	-6.32570	-6.23520	-0.10710
O	-6.14440	-5.82330	-1.41310
C	-7.08840	-6.44470	-2.26530
C	-7.91940	-7.30000	-1.46150
O	-7.08530	-6.20550	-3.45600
O	-5.62140	-5.82910	0.78080
C	-12.73260	-6.72560	-1.62470
C	-12.27680	-6.38690	-0.40720
C	-11.20150	-5.42990	-0.08340
C	-10.49460	-4.70200	-1.06240
C	-9.49000	-3.80440	-0.69960
C	-9.16130	-3.61370	0.64900
C	-9.85030	-4.33040	1.63170
C	-10.85920	-5.22690	1.26740
H	-7.16270	-8.16930	0.38020
H	-8.27120	-6.82180	0.62350
H	-8.72580	-7.89700	-1.86580
H	-13.53700	-7.44810	-1.73250
H	-12.33360	-6.30580	-2.54480
H	-12.73680	-6.85910	0.46160
H	-10.73240	-4.83200	-2.11430
H	-8.95900	-3.25260	-1.47140
H	-8.37650	-2.91520	0.92750
H	-9.60510	-4.19190	2.68180
H	-11.39300	-5.77750	2.03960

Styrene-maleic acid radical intermediate

Final energy = -766.009586 hartree

C	9.88450	0.80860	-4.93540
C	10.83110	0.96020	-3.75240
C	8.71480	3.18240	-5.01980
C	8.69650	1.72790	-4.90250
O	7.70560	3.80520	-5.35290
O	11.31050	2.03130	-3.39760
O	9.86250	3.85240	-4.79130
O	11.10670	-0.19460	-3.15280
C	8.27750	0.80860	1.63360
C	8.95950	-0.30280	1.31130
C	10.32720	-0.39110	0.76460
C	11.14800	0.73410	0.55210
C	12.43150	0.59290	0.02280
C	12.92830	-0.67680	-0.30800
C	12.12560	-1.80380	-0.09930

C	10.84200	-1.65950	0.43220
H	10.49410	1.01330	-5.83120
H	9.54770	-0.22710	-4.99800
H	7.70730	1.28340	-4.91570
H	10.53120	3.27690	-4.33630
H	11.71630	-0.04440	-2.39490
H	7.26760	0.74190	2.02860
H	8.68630	1.80950	1.51850
H	8.46880	-1.26560	1.45390
H	10.78580	1.72750	0.80060
H	13.04950	1.47390	-0.12980
H	13.93140	-0.78390	-0.71280
H	12.49910	-2.79380	-0.34860
H	10.22440	-2.54090	0.58980

Maleic anhydride-styrene radical intermediate

Final energy = -689.567014 hartree

C	1.32930	-6.95290	3.72470
C	2.08930	-6.69570	4.98660
C	3.27010	-5.92570	5.13350
C	3.91970	-5.25520	4.05110
C	5.07880	-4.51450	4.25660
C	5.64600	-4.40470	5.53610
C	5.02750	-5.05390	6.61840
C	3.86970	-5.79520	6.42650
C	6.28350	-8.39140	3.20070
C	7.27400	-7.35600	2.80760
O	8.17830	-7.20680	3.85930
C	7.80860	-8.08560	4.87720
C	6.60100	-8.82470	4.42630
O	8.42900	-8.15610	5.90530
O	7.36920	-6.70870	1.79840
H	1.38100	-8.01620	3.44120
H	1.68960	-6.36760	2.87420
H	0.26170	-6.72680	3.85960
H	1.69990	-7.16770	5.88800
H	3.50920	-5.32390	3.04830
H	5.55310	-4.02040	3.41180
H	6.55380	-3.82690	5.68740
H	5.45790	-4.97680	7.61430
H	3.40030	-6.29350	7.27240
H	5.47210	-8.69280	2.55130
H	6.11570	-9.57170	5.04090

Maleic acid-styrene radical intermediate

Final energy = -766.011508 hartree

C	-6.20860	-5.22780	0.81440
C	-5.34970	-6.37800	0.38710
C	-4.07070	-6.25450	-0.21440
C	-3.33490	-7.42100	-0.58670
C	-2.08170	-7.32600	-1.17780
C	-1.50340	-6.06900	-1.42380
C	-2.20540	-4.90710	-1.06690
C	-3.46180	-4.98970	-0.47490

C	-0.68990	-8.24000	3.59430
C	-0.55920	-8.87450	4.92610
C	-3.19410	-7.93160	3.78100
C	-1.86080	-7.81100	3.10730
O	-3.98360	-8.82490	3.53980
O	-1.48010	-9.10910	5.69270
O	-3.45620	-6.89730	4.59590
O	0.72520	-9.17950	5.20550
H	-6.45870	-4.56290	-0.02670
H	-7.14860	-5.58180	1.24810
H	-5.71120	-4.59710	1.56730
H	-5.72830	-7.38550	0.54710
H	-3.77470	-8.39840	-0.40000
H	-1.54570	-8.23190	-1.45170
H	-0.52290	-5.99750	-1.88700
H	-1.76460	-3.93080	-1.25470
H	-3.98750	-4.07760	-0.20730
H	0.21570	-8.13950	3.00470
H	-1.90360	-7.37090	2.11240
H	-4.35190	-7.01420	4.97400
H	0.76060	-9.59910	6.08810

Styrene-styrene radical

Final energy = -619.936054 hartree

C	1.14320	0.89560	-0.15760
C	1.28530	-0.50520	-0.66460
C	2.12920	-1.52250	-0.15160
C	2.94420	-1.37220	1.01290
C	3.76010	-2.40700	1.45730
C	3.80950	-3.63080	0.77050
C	3.01600	-3.80690	-0.37590
C	2.19330	-2.78340	-0.82550
C	-0.25770	1.24660	0.42570
C	-0.26920	2.70890	0.91690
C	-1.40850	0.99040	-0.54010
C	-2.54920	0.29600	-0.10750
C	-3.63750	0.08460	-0.96130
C	-3.60090	0.56220	-2.27470
C	-2.46990	1.25500	-2.72140
C	-1.38850	1.46880	-1.86170
H	1.88600	1.11410	0.61660
H	1.35460	1.59440	-0.98280
H	0.69190	-0.76530	-1.53940
H	2.92640	-0.43890	1.56760
H	4.36640	-2.26340	2.34870
H	4.45320	-4.43260	1.12190
H	3.04630	-4.74980	-0.91720
H	1.58450	-2.92910	-1.71530
H	-0.41510	0.59920	1.29840
H	-1.23140	2.95970	1.37820
H	0.52000	2.87560	1.66060
H	-0.10310	3.40330	0.08380
H	-2.58710	-0.08310	0.91240
H	-4.51020	-0.45480	-0.59990

H	-4.44190	0.39620	-2.94340
H	-2.42810	1.62880	-3.74210
H	-0.52120	2.01200	-2.23090

Styrene-maleic anhydride radical

Final energy = -689.585165 hartree

C	-5.81680	-7.44120	0.64400
C	-6.81000	-8.34470	-0.08520
O	-7.57910	-7.59180	-0.94960
C	-7.19610	-6.23170	-0.88840
C	-6.12600	-6.11170	0.06370
O	-7.76170	-5.40730	-1.57740
O	-6.96750	-9.53410	0.01700
C	-4.33300	-7.95830	0.53670
C	-3.84910	-8.03160	-0.91960
C	-3.42720	-7.15200	1.46030
C	-3.24040	-7.58230	2.78450
C	-2.43060	-6.86500	3.66920
C	-1.79010	-5.69690	3.24270
C	-1.96660	-5.25790	1.92710
C	-2.77720	-5.97910	1.04400
H	-6.08050	-7.46400	1.71330
H	-5.65760	-5.16730	0.30660
H	-4.37350	-8.98020	0.93010
H	-2.82130	-8.40800	-0.95330
H	-4.47350	-8.71770	-1.50290
H	-3.86850	-7.05680	-1.41850
H	-3.72940	-8.49350	3.12490
H	-2.29640	-7.22170	4.68750
H	-1.15730	-5.13760	3.92690
H	-1.47070	-4.35350	1.58280
H	-2.89120	-5.62010	0.02460

Styrene-maleic acid radical

Final energy = -766.032042 hartree

C	-6.27500	-6.27590	0.01580
C	-6.10600	-6.20150	1.53390
C	-7.63410	-8.10520	-1.16520
C	-6.49960	-7.70010	-0.36200
O	-8.51250	-7.35440	-1.58800
O	-5.20990	-6.73830	2.15780
C	-4.99100	-5.67150	-0.67760
C	-5.06590	-5.83070	-2.20620
C	-4.78190	-4.22160	-0.26240
C	-3.66350	-3.85950	0.50330
C	-3.44880	-2.52910	0.87960
C	-4.35570	-1.53730	0.49740
C	-5.47670	-1.88570	-0.26410
C	-5.68690	-3.21470	-0.63970
O	-7.63860	-9.44050	-1.42080
O	-7.07410	-5.47150	2.11790
H	-7.14740	-5.68550	-0.27240
H	-5.79280	-8.45980	-0.04240
H	-4.13770	-6.25400	-0.31280

H	-4.17660	-5.39000	-2.66890
H	-5.10620	-6.88830	-2.49080
H	-5.94800	-5.33350	-2.62460
H	-2.95180	-4.62450	0.80560
H	-2.57360	-2.27090	1.47060
H	-4.19190	-0.50290	0.78870
H	-6.18870	-1.12190	-0.56680
H	-6.56360	-3.46360	-1.23290
H	-8.42550	-9.64300	-1.96290
H	-6.91490	-5.45730	3.08350

Maleic anhydride-styrene radical

Final energy = -689.598048 hartree

C	0.05310	1.04420	-0.05710
C	1.69410	-0.84720	-0.38680
C	2.52910	-1.94080	-0.04250
C	3.45860	-2.45740	-0.99590
C	4.28610	-3.52940	-0.68810
C	4.22520	-4.13440	0.57870
C	3.31940	-3.64600	1.53290
C	2.48560	-2.57260	1.23700
C	0.99190	2.24970	-0.23000
C	0.20910	3.42690	0.30170
O	-0.94660	2.97140	0.92830
C	-1.06540	1.59100	0.81470
O	-1.97080	1.01200	1.35460
O	0.45650	4.60300	0.26280
C	0.67920	-0.23140	0.53090
H	-0.41750	0.79590	-1.01930
H	1.78020	-0.44760	-1.39560
H	3.51180	-1.99530	-1.97970
H	4.98400	-3.90190	-1.43390
H	4.87250	-4.97380	0.81720
H	3.26510	-4.11090	2.51430
H	1.78980	-2.21590	1.99120
H	1.90480	2.15320	0.37000
H	1.29440	2.44360	-1.26190
H	1.12990	0.00930	1.50580
H	-0.13200	-0.94430	0.74670

Maleic acid-styrene radical

Final energy = -766.044413 hartree

C	1.68450	1.82900	0.41160
C	2.91300	-0.37330	0.87180
C	2.84780	-1.74670	0.52810
C	2.57990	-2.22840	-0.79020
C	2.53010	-3.59180	-1.05890
C	2.74430	-4.53470	-0.04120
C	3.01070	-4.08770	1.26400
C	3.06130	-2.72970	1.54540
C	1.76640	3.09600	-0.44760
C	0.85040	4.20420	0.01820
C	0.30730	1.19390	0.31280
O	-0.34350	1.09870	-0.71440

O	0.08180	4.14220	0.96100
O	-0.11740	0.69090	1.48850
O	0.98050	5.31100	-0.74340
C	2.76680	0.80050	-0.04490
H	1.86820	2.08370	1.45960
H	3.11780	-0.13730	1.91560
H	2.40870	-1.52530	-1.59950
H	2.32230	-3.92830	-2.07170
H	2.70440	-5.59800	-0.26060
H	3.17890	-4.80840	2.06070
H	3.26850	-2.39350	2.55910
H	2.79160	3.48550	-0.43900
H	1.52910	2.87630	-1.49510
H	-0.98340	0.25910	1.34660
H	0.37280	5.99750	-0.40310
H	2.55560	0.49760	-1.07480
H	3.71910	1.35350	-0.07130

References

1 Barron, P. F., Hill, D. J. T., O'Donnell, J. H. & O'Sullivan, P. W. O. Applications of DEPT experiments to the carbon ^{13}C NMR of copolymers poly(styrene-co-maleic-anhydride). *Macromolecules* **17**, 6 (1984).

2 Ha, N. T. H. Determination of triad sequence distribution of copolymers of maleic anhydride and its derivates with donor monomers by ^{13}C N.M.R. spectroscopy. *Polymer* **40**, 6 (1999).
